# An Intervention to Reduce HIV Risk Behavior of Substance-Using Men Who Have Sex with Men: A Two-Group Randomized Trial with a Nonrandomized Third Group

**DOI:** 10.1371/journal.pmed.1000329

**Published:** 2010-08-24

**Authors:** Gordon Mansergh, Beryl A. Koblin, David J. McKirnan, Sharon M. Hudson, Stephen A. Flores, Ryan E. Wiegand, David W. Purcell, Grant N. Colfax, for the Project MIX Study Team

**Affiliations:** 1 Division of HIV/AIDS Prevention, Centers for Disease Control and Prevention, Atlanta, Georgia, United States of America; 2 New York Blood Center, New York, New York, United States of America; 3 University of Illinois at Chicago and Howard Brown Health Center, Chicago, Illinois, United States of America; 4 Health Research Association, Los Angeles, California, United States of America; 5 San Francisco Department of Public Health, San Francisco, California, United States of America; University of Connecticut, United States of America

## Abstract

**Background:**

Substance use during sex is associated with sexual risk behavior among men who have sex with men (MSM), and MSM continue to be the group at highest risk for incident HIV in the United States. The objective of this study is to test the efficacy of a group-based, cognitive-behavioral intervention to reduce risk behavior of substance-using MSM, compared to a randomized attention-control group and a nonrandomized standard HIV-testing group.

**Methods and Findings:**

Participants (*n* = 1,686) were enrolled in Chicago, Los Angeles, New York City, and San Francisco and randomized to a cognitive-behavioral intervention or attention-control comparison. The nonrandomized group received standard HIV counseling and testing. Intervention group participants received six 2-h group sessions focused on reducing substance use and sexual risk behavior. Attention-control group participants received six 2-h group sessions of videos and discussion of MSM community issues unrelated to substance use, sexual risk, and HIV/AIDS. All three groups received HIV counseling and testing at baseline. The sample reported high-risk behavior during the past 3 mo prior to their baseline visit: 67% reported unprotected anal sex, and 77% reported substance use during their most recent anal sex encounter with a nonprimary partner. The three groups significantly (*p*<0.05) reduced risk behavior (e.g., unprotected anal sex reduced by 32% at 12-mo follow-up), but were not different (*p*>0.05) from each other at 3-, 6-, and 12-mo follow-up. Outcomes for the 2-arm comparisons were not significantly different at 12-mo follow-up (e.g., unprotected anal sex, odds ratio = 1.14, confidence interval = 0.86–1.51), nor at earlier time points. Similar results were found for each outcome variable in both 2- and 3-arm comparisons.

**Conclusions:**

These results for reducing sexual risk behavior of substance-using MSM are consistent with results of intervention trials for other populations, which collectively suggest critical challenges for the field of HIV behavioral interventions. Several mechanisms may contribute to statistically indistinguishable reductions in risk outcomes by trial group. More explicit debate is needed in the behavioral intervention field about appropriate scientific designs and methods. As HIV prevention increasingly competes for behavior-change attention alongside other “chronic” diseases and mental health issues, new approaches may better resonate with at-risk groups.

**Trial Registration:**

ClinicalTrials.gov NCT00153361

*Please see later in the article for the Editors' Summary*

## Abstract 2

In a randomized trial of a behavioral intervention among substance-using men who have sex with men, aimed at reducing sexual risk behavior, Mansergh and colleagues fail to find evidence of a reduction in risk from the intervention.

## Editors' Summary

**Background:**

AIDS first emerged in the early 1980s among gay men living in the US. As the disease spread around the world, it became clear that AIDS also affects heterosexual men and women. Now, three decades on, more than 30 million people are infected with HIV, the virus that causes AIDS. HIV is most often spread by having unprotected sex with an infected partner and, globally, most sexual transmission of HIV now occurs during heterosexual sex. However, 5%–10% of all new HIV infections still occur in men who have sex with men (MSM, a term that encompasses gay, bisexual, transgendered, and heterosexual men who sometimes have sex with men) and, in several high-income countries, male-to-male sexual contact remains the most important HIV transmission route. In the US, for example, more than half of the approximately 50,000 people who become infected with HIV every year do so through male-to-male sexual contact.

**Why Was This Study Done?:**

In countries where MSM are the group at highest risk of HIV infection, any intervention that reduces HIV transmission in MSM should have a major effect on the overall HIV infection rate. Among MSM, sexual behaviors that increase the risk of HIV infection (for example, not using a condom, having anal sex, having sex with a partner of unknown HIV status, and having sex with many partners) are associated with the use of alcohol and noninjection drugs (for example, inhaled amyl nitrite or poppers) during or shortly before sexual encounters. In this study (Project MIX), the researchers investigate whether a group-based behavioral intervention reduces sexual risk behavior in substance-using MSM.

**What Did the Researchers Do and Find?:**

The researchers recruited substance-using MSM from four US cities who had had risky sex at least once in the past 6 months. Participants were randomized to a cognitive-behavioral intervention or to an attention-control group; a third, nonrandomized group of MSM formed a standard HIV counseling and testing only group. All the groups had HIV counseling and testing at the start of the study and completed a questionnaire about their substance use and sexual risk behavior during their most recent anal sex encounter. The cognitive-behavior group then received six weekly 2-hour group sessions focused on reducing substance use and sexual risk behavior by helping the men change their thinking (cognition) and behavior regarding sexual risk taking. The attention-control group received six group sessions about general MSM issues such as relationships, excluding discussion of substance use, and sexual risk behavior. The participants in both of these groups completed the questionnaire about their substance use and sexual risk behavior again at 3, 6, and 12 months after the group sessions; the participants in the standard HIV counseling and testing group completed the questionnaire again about 5 months after completing the first questionnaire (to control for the time taken by the other two groups to complete the intervention). At baseline, about 67% of the participants reported unprotected anal sex and 77% reported substance use during their most recent anal sex encounter with a nonprimary partner. At the 3-month follow-up, the incidence of sexual risk behavior had fallen to about 43% in all three groups; the incidence of substance use during sex had fallen to about 50%. Risk taking and substance use remained at these levels in the intervention and attention-control groups at the later follow-up time points.

**What Do These Findings Mean?:**

These findings suggest that this cognitive-behavioral intervention is no better at reducing sexual risk taking among substance-using MSM than is an unrelated video-discussion group or standard HIV counseling and testing. One explanation for this negative result might be that brief counseling is especially effective with people who are ready for a change such as MSM willing to enroll in an intervention trial of this type. Alternatively, just being in the trial might have encouraged all the participants to self-report reduced risk behavior. Thus, alternative scientific designs and methods might be needed to find behavioral interventions that can effectively reduce HIV transmission among substance-using MSM and other people at high risk of HIV infection. Importantly, however, these findings raise the question of whether more extensive, multilevel interventions or broader lifestyle and positive health approaches (rather than single-level or single-subject behavioral interventions) might be needed to reduce sexual risk behavior among substance-using MSM.

**Additional Information:**

Please access these Web sites via the online version of this summary at http://dx.doi.org/10.1371/journal.pmed.1000329.

- Information is available from the US Department of Health and Human Services on HIV prevention programs, research, and policy
- Information is available from the US National Institute of Allergy and Infectious Diseases on HIV infection and AIDS
- HIV InSite has comprehensive information on all aspects of HIV/AIDS, including information on HIV transmission and transmission in gay men and other MSM, on substance abuse and HIV/AIDS, and on safer sex
- Information is available from Avert, an international AIDS nonprofit, on all aspects of HIV/AIDS, including information on HIV, AIDS, and men who have sex with men and on drink, drugs, and sex (in English and Spanish)
- The US Centers for Disease Control and Prevention also have information for the public and for professionals about HIV/AIDS among men who have sex with men (in English and Spanish)
- The US National Institute on Drug Abuse has information on HIV/AIDS and drug abuse, including a resource aimed at educating teenagers about the link between drug abuse and the spread of HIV in the US (in English and Spanish)

## Introduction

Men who have sex with men (MSM) continue to be the largest group newly HIV infected each year in the United States [1]. Alcohol and noninjection substance use is associated with sexual risk behavior in this population [2]–[8], and sexual risk increases when substances are used soon before or during sexual encounters [4],[9]. Thus, substance-using MSM are likely to be contributing disproportionately to HIV incidence in the US [10]. Although interventions have been tested with substance-abusing MSM in drug treatment settings [11]–[13], few interventions have been tested with substance-using MSM not in treatment [14]. This study is, to our knowledge, the first large, multicity, randomized intervention trial specifically addressing sexual risk among out-of-treatment substance-using MSM.

Randomized trials of HIV risk behavioral interventions [15]–[18]—particularly for MSM—have generally used two-group designs: an attention-control group (e.g., content materials unrelated to intervention content) or a standard group (e.g., HIV counseling and testing), but rarely both. These interventions generally found no difference between the two groups in HIV risk outcomes [15]–[18]. An ideal approach is a three-group trial that distinguishes the effects of content and attention. We used a modified three-group design: randomized intervention and attention-control groups and a nonrandomized standard group. The research objective was to systematically test the efficacy of a group-based, cognitive-behavioral intervention to reduce sexual risk of substance-using MSM.

## Methods

The protocol (Text S1) was approved by institutional review boards at each of the local sites and the Centers for Disease Control and Prevention (CDC). Details of the baseline assessment have been published [2]. The CONSORT requirements (Text S2) were completed as requested.

### Study Population

Participants for all three groups were recruited and assessed through follow-up in Chicago, Los Angeles, New York City, and San Francisco, from October 2004 through April 2008, through street and MSM venue outreach, agency/business-based posters and flyers, ads in print media, and word of mouth. Each city tailored its recruitment campaigns to the local population. Standard power analysis calculations were conducted a priori to determine the desired sample size, based on 80% statistical power to detect a 25% reduction in risk behavior, and an expected 80% retention across follow-up waves.

Men were eligible to participate if they reported (1) being drunk or “buzzed” on alcohol two or more times, or high on noninjection drugs at least once, during (or 2 h before) anal sex in the past 6 mo, and (2) at least one unprotected anal sex episode in the past 6 mo with a male partner whose HIV serostatus was unknown or different from their own. Men were ineligible if they (1) reported only marijuana or use of erectile dysfunction medications soon before or during anal sex in the past 6 mo; (2) reported injecting drugs other than steroids, hormones, prescribed medications, or methamphetamine in the past 6 mo; (3) had known for less than 6 mo that they were HIV-positive; or (4) were currently participating in another HIV behavioral intervention trial. For the two randomized groups, eligible men agreed to participate in a six-session, group-based intervention and to complete assessments at baseline and at 3-, 6-, and 12-mo follow-up waves. For the standard group, eligible men agreed to complete an assessment at baseline and at 3-mo follow-up.

### Design and Procedures

#### Screening

Potential participants were initially screened by telephone. Those who were eligible and willing to participate were scheduled for a baseline appointment; HIV-positive men were asked to bring documentation of their serostatus (e.g., HIV-positive test result or treatment prescription) to the appointment.

#### Baseline assessment

At the baseline assessment, men were rescreened for eligibility. If eligible, the men provided written informed consent and completed an audio computer-assisted self-interview (ACASI). All men received standard HIV risk-reduction counseling [19]; HIV-negative and unknown-serostatus men and HIV-positive men without documentation of their serostatus were administered a rapid HIV test. Contact information was collected, and the next appointment was scheduled (i.e., group randomization for the intervention and attention-control groups; and 3-mo follow-up for the standard group). Lastly, the men were reimbursed for their time and travel, as determined by each site (range, US$25–US$40). The assessment collected information on demographic characteristics, substance use, and sexual risk behavior during the participant's most recent anal sex encounter, and psychosocial and mental health measures. We took steps to minimize bias, including the use of different staff members for group activities versus assessment. Perhaps more important, behavior was assessed in a private location by the use of ACASI.

#### Randomization

Participants were randomized by laptop computer program to intervention and attention-control groups upon arrival at the first group session. A minimum of ten (five per group) and a maximum of 20 (ten per group) men were needed for randomization. On-site computerized randomization was blocked by HIV serostatus so that if possible, the intervention and the attention-control group each contained at least two men who were HIV-positive and at least two men who were HIV-negative (participant code and blocking information were pre-entered into the computer prior to the first session). Each group was also blocked for a minimum of five participants. Randomization stopped after 20; men who arrived later were reimbursed US$15 for transportation costs and if possible, rescheduled for another randomization session.

Because of insufficient funding to fully randomize all three groups, enrollment in the standard group took place immediately after enrollment in the intervention and attention-control groups had been completed. Even though enrollment for the two randomized immediately preceded enrollment for the nonrandomized control group, follow-up assessment overlapped for the three groups.

#### Group sessions

Intervention and attention-control groups consisted of six weekly 2-h group sessions, facilitated by trained staff (facilitator protocols available from G. Mansergh, on request). Intervention content consisted of cognitive-behavioral techniques and relevant skills building [20],[21], including modeling and behavioral rehearsal. Specific intervention modules helped participants analyze their substance use and sexual risk patterns, identify situational triggers for risky behavior, develop behavioral alternatives and negotiation strategies, and plan for change. Behavior change attempts over the 6 wk allowed for feedback and positive reinforcement on a weekly basis. Intervention content was based on formative research and pilot testing. A 10-min break occurred in the middle of each weekly 2-h session.

The modules of the attention-control group consisted of videos, and group discussion was focused on MSM-related issues unrelated to substance use, sexual risk behavior, and HIV, such as relationships, spirituality, and racism. Twelve 45- to 55-min modules each consisted of a 20- to 30-min video followed by discussion of the video; two modules were presented each meeting of the 6 wk, with a 10-min break in the middle of each night.

Staff members were extensively trained to facilitate the intervention and attention-control materials according to the facilitator manuals. For the intervention group, staff were trained to lead intervention exercises and discussions, emphasizing primary messages. For the attention-control group, staff were trained to subtly redirect discussion—away from substance use, sexual risk behavior, and HIV/AIDS. Intervention and attention-control group sessions were taped, and two of the six sessions for each group were reviewed and scored to ensure that the material in the facilitator manuals was covered. For the intervention group, adherence to the materials during the six sessions averaged 94%. For the attention-control group, intervention content (alcohol or drug use, sexual risk, and HIV/AIDS) discussion was to be avoided; unintended discussion of topics related to HIV, substance use, or sexual risk behaviors occurred in 3% of the sessions and were redirected by facilitators.

#### Follow-up assessment

For the intervention and attention-control groups, follow-up assessment waves took place 3, 6, and 12 mo after the final group session. In follow-up sessions, participants completed the same ACASI behavioral assessment as at baseline, updated their contact information, were reimbursed for time and travel (increasing at each follow-up, ranging from US$25–US$50), and received an appointment for the next follow-up. The 12-mo follow-up included HIV testing for HIV-negative participants and counseling for all participants. For the standard group, follow-up assessment took place at 3 mo plus 7 wk (to control for lag time because of completion of sessions in the other two groups).

### Statistical Analysis

The level of significance for all tests was set at *p*<0.05. Sample size was based on 80% statistical power to detect an approximate 25% change in behavior (e.g., unprotected anal sex) from baseline to follow-up, which is consistent with findings from meta-analyses of HIV behavioral intervention trials [22],[23]. Bivariate comparisons of outcomes and predictors at each follow-up wave were performed with chi-square tests; 95% confidence intervals (CIs) for raw proportions were calculated with asymptotic standard errors and a continuity correction.

The primary outcomes were six dichotomous variables, all focused on participant behavior during the most recent anal sex encounter with a nonprimary partner: (1) unprotected anal sex; (2) unprotected anal sex with a discordant partner (i.e., a partner whose HIV serostatus was unknown or different from that of the participant); (3) alcohol use soon before or during unprotected anal sex; (4) alcohol use during or before unprotected anal sex with a discordant partner; (5) drug use soon before or during unprotected anal sex; and (6) drug use soon before or during unprotected anal sex with a discordant partner. Three secondary outcomes of interest were (1) unprotected receptive anal sex, (2) unprotected insertive anal sex, and (3) substance (alcohol or drug) use soon before or during anal sex, whether protected or unprotected anal sex.

For longitudinal analyses, we used a generalized linear mixed model to evaluate the dichotomous outcomes. A random intercept for each participant was incorporated into the model to control for any correlation within participants in the four follow-up waves. The set of covariates consisted of site (Chicago, Los Angeles, New York, San Francisco), age (18–24, 25–34, 35–44, ≥45 y), education level (high school diploma or less, some post-high school training, college degree or more), primary race or ethnicity (black, Hispanic/Latino, white, other), self-identified as gay/homosexual (bisexual/other), self-reported baseline HIV serostatus, assessment wave (baseline: 3-, 6-, and 12-mo follow-up), and trial group (intervention, attention-control, standard group; blinded in analysis), as well as an interaction between follow-up wave and trial group to test for efficacy.

A generalization of the Satterthwaite approximation [24] was used to adjust the degrees of freedom. We used a multiple imputation approach [25] with adaptive rounding for binary variables [26] to impute missing outcome variables. Ten imputations were aggregated for the results and drug use covariates were incorporated into the imputation procedure to increase the efficiency of the imputed observations [27]. Models were also run on the raw, nonimputed data. Inferences for the trial arm, wave, and interaction between trial arm and wave did not differ between the analyses of the raw and multiply imputed data. Rates of reduction were calculated from population-averaged rates, which control for all other covariates in the multivariable model. Models were calculated by using the GLIMMIX and MIANALYZE procedures in Statistical Analysis Software (SAS), version 9.2 (SAS Institute, Inc.), and model fit was evaluated by diagnostic statistics and residual plots.

## Results

### Sample Characteristics

In total, 1,686 men were enrolled: 1,206 were randomly assigned to the intervention and attention-control groups; 480 were assigned to the standard group (Table 1). The sample was diverse in terms of age, race/ethnicity, baseline self-reported HIV serostatus, and education level. Nearly one in six men did not identify themselves as gay or homosexual. There were no differences (*p*>0.05) in descriptive characteristics for the two randomized groups. Participants in the standard group were younger and more educated, and fewer were black or HIV-positive (*p*<0.05).

**Table 1:** Project MIX, baseline characteristics and bivariate comparisons, by group, 2004–2008.

Characteristic		Intervention (*n* = 599)	Attention Control (*n* = 607)	Standard (*n* = 480)
		*n* (%)	*n* (%)	*n* (%)
Age group (y)	18–24	70 (12)	56 (9)	64 (13)^a^
	25–34	158 (26)	162 (27)	150 (31)
	35–44	239 (40)	265 (44)	168 (35)
	≤45	132 (22)	124 (20)	98 (21)
Primary race/ethnicity	Black	200 (33)	194 (32)	124 (26)^a^
	Hispanic/Latino	120 (20)	102 (17)	97 (20)
	White	225 (38)	241 (40)	216 (45)
	Other	54 (9)	70 (11)	43 (9)
Education level	≤High school diploma	190 (32)	191 (31)	111 (23)^a^
	>High school education	198 (33)	216 (36)	179 (37)
	≥College degree	211 (35)	200 (33)	190 (40)
HIV serostatus	Positive	307 (51)	300 (49)	178 (37)^a^
	Negative	248 (42)	272 (45)	245 (51)
	Unknown	44 (7)	35 (6)	57 (12)
Sexual orientation identification	Gay/homosexual	501 (84)	508 (84)	410 (85)
	Bisexual/other	98 (16)	99 (16)	70 (15)
City	Chicago	159 (27)	160 (26)	150 (31)
	Los Angeles	118 (20)	118 (20)	99 (21)
	New York	151 (25)	159 (26)	102 (21)
	San Francisco	171 (28)	170 (28)	129 (27)

a Standard group significantly different from intervention and attention-control groups (chi-square test, *p*<0.05); other overall 3-way tests were nonsignificant (*p*>0.05).

### Enrollment and Retention

**Figure 1:**
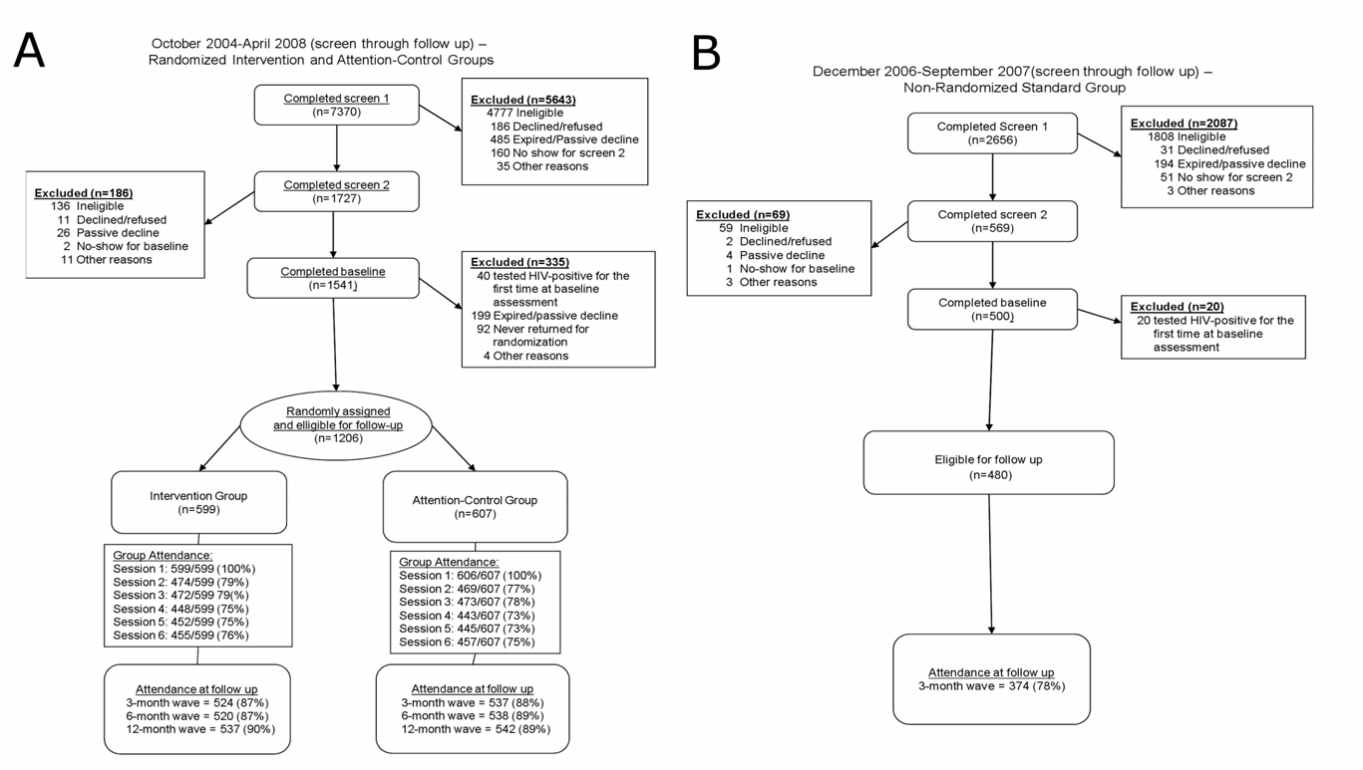
CONSORT flow diagrams. (A) October 2004–April 2008 (screen through 3, 6, and 12-mo follow-up): randomized intervention and attention-control groups. (B) December 2006–September 2007 (screen through 3-mo follow-up): nonrandomized standard control group.

For randomization of the intervention and attention-control groups, 7,370 men were screened, and 1,206 were enrolled at the randomization session (*n* = 599 in intervention group; *n* = 607 in attention-control group; Figure 1). A total of 2,656 men were screened for the standard group to achieve enrollment of 480.

Session attendance and retention were high throughout the trial (Figure 1). Session attendance for the two randomized groups did not differ (*p*>0.05): attendance at each session after session 1 ranged from 73% to 79%. Retention at follow-up for these groups also did not differ (*p*>0.05): retention at each follow-up wave ranged from 87% to 90%. For the standard group, retention at 3-mo follow-up was lower: 78%.

Attrition analysis of follow-up waves for the two randomized groups found no differences (*p*>0.05) in main effects of follow-up wave and group, or in interaction effects of follow-up wave by group. Further, unprotected anal sex and discordant unprotected anal sex were not associated (*p*>0.05) with loss to follow-up. Attrition analysis at 3-mo follow-up for the three groups found greater (*p*<0.05) loss to follow-up in the nonrandomized group than in the randomized groups. Again, unprotected anal sex and discordant unprotected anal sex were not associated with attrition. In comparisons by age and education, more of the youngest participants (18–24 y versus ≥45 y) and those with the least education (high school diploma or less versus college degree or higher) were missing at 3-mo follow-up (*p*<0.05).

### Baseline Behavioral Characteristics

At baseline, two-thirds of the sample reported that they had not used protection during their most recent anal sex encounter (Table 2). Two of every five men reported discordant unprotected anal sex. More than three-fourths of the sample reported at baseline that they had used alcohol or drugs soon before or during their most recent anal sex encounter: 57% reported alcohol use, and 56% reported drug use (unpublished data). Over 40% of the sample reported alcohol use, and over 40% reported drug use before having unprotected sex in their most recent anal sex encounter; 25% reported alcohol and 25% reported drug use before having discordant unprotected sex in that encounter. The three groups did not differ at baseline on any of these risk behaviors (Table 2).

**Table 2:** Project MIX primary and secondary outcome behaviors, by group at assessment wave, 2004–2008.

Behavior Soon Before or During Most Recent Anal Sex Encounter	Baseline (%)			3 mo (%)			6 mo (%)		12 mo (%)	
	Interv (*n* = 599)	Attn (*n* = 607)	Stand (*n* = 480)	Interv (*n* = 524)	Attn (*n* = 537)	Stand (*n* = 374)	Interv (*n* = 520)	Attn (*n* = 538)	Interv (*n* = 537)	Attn (*n* = 542)
**Primary outcome**										
UA	67	68	68	43	44	43	43	42	40	38
DUA	39	38	43	19	21	23	17	18	18	20
Substance use and UA										
Alcohol	42	40	42	21	21	21	20	21	17	17
Drugs	45	43	39	26	24	22	27	23	22	19
Substance use and DUA										
Alcohol	27	23	28	10	11	13	8	9	9	10
Drugs	25	24	24	12	12	11	10	9	10	11
**Secondary outcome**										
URA	38	41	39	24	26	25	23	24	25	22
UIA	39	36	35	24	22	21	24	23	20	20
Substance use	77	78	75	54	52	53	52	49	46	44

Attn, attention-control group; Interv, intervention group; Stand, standard group; URA, unprotected receptive anal sex; UIA, unprotected insertive anal sex.

### Study Outcomes

Risky behavior was lower at 3-, 6-, and 12-mo follow-up waves for the primary and secondary outcome variables in all trial groups (Table 2). In multivariate analysis, controlled for demographic variables (Table 3), each of the primary outcomes significantly (*p*<0.05) decreased at 3-mo follow-up. These reductions were maintained at 6- and 12-mo follow-up for the intervention and attention-control groups. For example, in the 2-arm comparison, the population-averaged means indicated unprotected anal intercourse in the prior 3 mo reduced by 32% at 12-mo (27% at 3-mo and 29% at 6-mo) follow-up relative to the baseline rate; the reductions did not differ by follow-up wave (*p*>0.05). After standardizing the reductions, similar results were found for the other outcome variables at each of the follow-up time points in 2-arm comparisons, and at 3-mo follow-up for 3-arm comparisons (Table 3). The pattern was similar for the secondary outcome variables: unprotected receptive sex, unprotected insertive sex, and substance use before anal sex. Multivariate analysis found no differences (Table 3) between the randomized intervention and attention-control group for outcome variables at each of the follow-up waves. For example, the outcomes for the 2-arm comparison were not significantly different at 12-mo follow-up (e.g., unprotected anal [UA] sex, odds ratio [OR] = 1.14, 95% CI = 0.86–1.51; HIV-discordant UA [DUA], OR = 0.92, CI = 0.66–1.28; alcohol UA, OR = 1.06, CI = 0.75–1.49; drug UA, OR = 1.21, CI = 0.86–1.70; alcohol DUA, OR = 0.86, CI = 0.56–1.32; drug DUA, OR = 0.98, CI = 0.64–1.48). Further, there were no differences in any of the outcomes between all three groups at 3-mo follow-up (Table 3). The pattern of results was notably consistent for the 18 analysis models presented in Table 3, and thus likely not due to chance.

**Table 3:** Project MIX, multivariate results for outcome behaviors, by group at assessment wave, 2004–2008.

Group	Primary Outcomes, AOR (CI)						Secondary Outcomes, AOR (CI)		
	UA	DUA	Alcohol-UA	Drug-UA	Alcohol-DUA	Drug-DUA	URA	UIA	Substance Use during Sex^a^
**2-Group analysis for 3-, 6-, 12-mo follow-up waves (** ***n*** ** = 1,206)**									
Attention-control (referent)									
Intervention	0.95 (0.72–1.26)	1.06 (0.82–1.38)	1.15 (0.88–1.50)	1.07 (0.81–1.42)	1.25 (0.93–1.67)	1.04 (0.77–1.40)	0.85 (0.64–1.13)	1.15 (0.87–1.52)	0.96 (0.70–1.30)
Follow-up (baseline, referent)									
3 mo	0.34 (0.26–0.43)	0.41 (0.31–0.54)	0.37 (0.28–0.49)	0.36 (0.27–0.47)	0.40 (0.29–0.57)	0.42 (0.30–0.58)	0.46 (0.35–0.61)	0.48 (0.36–0.63)	0.27 (0.21–0.36)
6 mo	0.29 (0.23–0.38)	0.34 (0.25–0.45)	0.37 (0.28–0.49)	0.32 (0.24–0.42)	0.32 (0.23–0.45)	0.28 (0.19–0.40)	0.40 (0.30–0.53)	0.50 (0.37–0.66)	0.24 (0.18–0.31)
12 mo	0.24 (0.18–0.31)	0.37 (0.28–0.49)	0.27 (0.20–0.36)	0.25 (0.18–0.33)	0.34 (0.24–0.48)	0.33 (0.24–0.47)	0.33 (0.25–0.43)	0.40 (0.31–0.54)	0.19 (0.14–0.25)
Intervention group comparisons									
Baseline—Interv (versus Attn)	0.95 (0.71–1.27)	1.06 (0.81–1.39)	1.15 (0.88–1.51)	1.07 (0.80–1.43)	1.25 (0.92–1.69)	1.04 (0.76–1.41)	0.85 (0.63–1.14)	1.15 (0.86–1.53)	0.96 (0.69–1.32)
3 mo—Interv (versus Attn)	0.93 (0.70–1.22)	0.89 (0.64–1.23)	0.99 (0.72–1.37)	1.06 (0.77–1.46)	0.88 (0.58–1.32)	0.92 (0.62–1.37)	0.86 (0.63–1.19)	1.11 (0.81–1.53)	1.13 (0.85–1.49)
6 mo—Interv (versus Attn)	1.01 (0.76–1.33)	0.89 (0.64–1.25)	0.94 (0.68–1.30)	1.26 (0.91–1.75)	0.87 (0.56–1.35)	1.14 (0.74–1.76)	0.94 (0.67–1.30)	1.03 (0.75–1.42)	1.12 (0.85–1.49)
12-mo—Interv (versus Attn)	1.14 (0.86–1.51)	0.92 (0.66–1.28)	1.06 (0.75–1.49)	1.21 (0.86–1.70)	0.86 (0.56–1.32)	0.98 (0.64–1.48)	1.27 (0.91–1.77)	1.05 (0.75–1.47)	1.10 (0.83–1.45)
**3-Group analysis for 3-mo follow-up waves (** ***n*** ** = 1,686)**									
Standard (referent)									
Intervention	0.93 (0.71–1.23)	0.87 (0.67–1.13)	1.00 (0.77–1.31)	1.13 (0.86–1.48)	0.97 (0.73–1.30)	1.00 (0.74–1.36)	0.84 (0.64–1.12)	1.17 (0.89–1.54)	1.04 (0.77–1.40)
Attention-control	0.97 (0.74–1.28)	0.84 (0.64–1.09)	0.88 (0.68–1.15)	1.05 (0.80–1.38)	0.80 (0.59–1.07)	0.97 (0.71–1.31)	0.98 (0.74–1.30)	1.03 (0.78–1.35)	1.09 (0.81–1.48)
Follow-up (baseline, referent)									
3-mo	0.34 (0.26–0.45)	0.39 (0.29–0.53)	0.35 (0.26–0.47)	0.39 (0.29–0.53)	0.35 (0.25–0.5)	0.39 (0.27–0.57)	0.52 (0.37–0.72)	0.50 (0.36–0.69)	0.37 (0.27–0.49)
Intervention group comparisons									
Baseline—Interv (versus Attn)	0.96 (0.73–1.26)	1.05 (0.81–1.35)	1.14 (0.88–1.47)	1.07 (0.82–1.39)	1.22 (0.91–1.62)	1.04 (0.77–1.39)	0.86 (0.65–1.13)	1.14 (0.88–1.49)	0.95 (0.70–1.28)
Baseline—Interv (versus Stand)	0.93 (0.70–1.24)	0.87 (0.67–1.14)	1.00 (0.77–1.32)	1.13 (0.85–1.50)	0.97 (0.72–1.31)	1.00 (0.73–1.37)	0.84 (0.63–1.13)	1.17 (0.88–1.56)	1.04 (0.76–1.42)
Baseline—Attn (versus Stand)	0.97 (0.73–1.30)	0.84 (0.64–1.09)	0.88 (0.67–1.16)	1.05 (0.79–1.39)	0.80 (0.59–1.08)	0.97 (0.70–1.32)	0.98 (0.74–1.32)	1.03 (0.77–1.37)	1.09 (0.80–1.50)
3 mo—Interv (versus Attn)	0.95 (0.73–1.23)	0.88 (0.65–1.20)	1.00 (0.74–1.36)	1.06 (0.79–1.42)	0.86 (0.58–1.27)	0.93 (0.64–1.36)	0.88 (0.65–1.19)	1.15 (0.85–1.54)	1.12 (0.87–1.45)
3 mo—Interv (versus Stand)	0.95 (0.72–1.25)	0.78 (0.57–1.08)	0.98 (0.71–1.36)	1.09 (0.79–1.51)	0.79 (0.53–1.19)	0.96 (0.63–1.45)	0.80 (0.58–1.11)	1.18 (0.86–1.63)	0.98 (0.74–1.29)
3 mo—Attn (versus Stand)	0.99 (0.75–1.31)	0.89 (0.65–1.22)	0.98 (0.71–1.36)	1.03 (0.74–1.43)	0.93 (0.62–1.38)	1.03 (0.69–1.55)	0.91 (0.66–1.26)	1.03 (0.75–1.42)	0.87 (0.67–1.15)

Controlled for all variables listed plus city, age, race/ethnicity, education level, sexual orientation identification, and HIV serostatus.

AOR, adjusted odds ratio; Attn, attention-control group; Interv, intervention group; Stand, standard of care group; UIA, unprotected insertive anal sex; URA, unprotected receptive anal sex.

a Substance use soon before or during most recent anal sex encounter.

## Discussion

In this cognitive-behavioral intervention trial, the three groups significantly reduced risk behavior to similar levels at each follow-up time point (e.g., overall 32% reduction in UA at 12-mo follow-up) and were not different from one another. These trial results for reducing risk behavior of substance-using MSM are consistent with results of other randomized intervention trials for MSM and substance-using populations [15]–[18], which collectively point to critical challenges for the field of HIV behavioral interventions and perhaps interventions for other health behaviors as well. For example, a systematic review of multibehavior interventions to reduce risk for coronary heart disease [28] found that the interventions had little or no impact on risk of heart disease, and only small reductions in more proximal indicators (e.g., salt intake, cholesterol, and blood pressure levels). The authors concluded that although numerous studies have been done, attempts at reduction in behavior-related risk factors for heart disease have limited success. Perhaps more intensive, multilevel interventions (i.e., interventions that address more than one level, including individual, small group, community, and structural levels) are needed to provide preferential results compared to control groups [29]; interventions that focus on more structural and policy level interventions (e.g., free and accessible condoms) could provide broader and more impactful behavior change [29]. Such an approach to behavioral interventions for substance-using MSM may be warranted.

Risk reduction at follow-up waves for intervention and comparison groups in this trial may be similar for several reasons. First, regression to the mean may account for behavioral risk reduction at follow-up for all groups [30]. Second, perhaps standard HIV counseling and testing are adequate to reduce risk, and more intensive interventions provide no additional benefit. In fact, some studies have found HIV testing to reduce measures of risk [19], as have studies of brief counseling [20],[21]. Brief counseling may be especially effective with people ready for change, as in persons willing to enroll in an intervention trial such as ours.

Another possible mechanism for reported risk reduction across groups is that a trial's unintentional “demand” for change (through the psychosocial dynamics of selective recall and social desirability) reduces reports of risky behavior but does not reduce the risky behavior itself. We did not find a relationship between a standard measure of social desirability and risk reports in this cohort, although general social desirability measures may not accurately assess the dynamic in the context of this study. A well-designed methodological study would have to examine potential mediators of real and reported behavior change; for example, including a post-test–only intervention condition and an assessment-only condition (e.g., the Solomon Four-Group Design) could test this approach. During repeated assessments, some men may learn (and choose) to complete their follow-up assessment more quickly by reporting less risk, although we did not find this to be a clear pattern in our trial.

Our trial had several limitations, including standard concerns in behavioral research regarding self-report (although we did use ACASI to minimize this bias) [31], and behavioral regression to the mean over time, as mentioned [30]; this may especially be the case with the very high-risk enrollment criteria in this study (i.e., greater potential for regression to the mean at follow-up relative to less-risky samples). Not all of the outcome variables are entirely exclusive from one another (e.g., UA with a discordant partner is subsumed in UA overall). Although the intervention and attention-control groups were randomized, the standard group was not: because of funding restrictions, enrollment for the standard group took place after enrollment for the other groups, and this group provided only a 3-mo follow-up. Although a few demographic differences were noted between the standard group and the other groups at baseline, baseline risk behavior did not differ; we controlled for baseline demographic factors in outcome analyses, and there were no group differences.

Future methodological studies should systematically assess effects of behavioral intervention methods, including potential change mechanisms noted above, which could inform other areas of health behavior research as well as HIV prevention, particularly in the context of multilevel interventions. If recommended counseling and testing [19] constitute an acceptable standard for reducing risk behavior, then perhaps this type of counseling and testing is an appropriate comparison group in trials, especially given that expensive attention-control groups prohibit inclusion of other methodologically important groups (e.g., assessment only; post-test only). More explicit debate is needed in the HIV behavioral intervention field about appropriate study methods and designs, and new paradigms should be explored.

Alcohol- and drug-using MSM contribute to HIV incidence among US MSM, and they are a critical group for focused risk reduction [32]; this is one of the first and the largest intervention trials tested on this high-risk population to our knowledge. To achieve behavior change beyond that of standard HIV counseling and testing, new approaches should be considered. Colleagues have suggested a focus on “syndemics” of HIV, substance use, depression, etc. [33], and broader perspectives on health and healthy lifestyles beyond HIV. Similarly, “positive psychology” and a focus on health strengths is an emerging direction for the field of health research [34]. Holistic approaches such as these may increasingly resonate, as HIV prevention competes more and more for behavior-change attention alongside traditional chronic diseases and mental health issues [35]. Other possible directions for future research include a focus on environmental factors that affect sexual risk behavior of substance-using MSM, and enhancing behavioral uptake and adherence of promising biomedical interventions for high-risk MSM [35], such as pre- and post-exposure prophylaxis.

## Supporting Information

Text S1 Trial protocol.
(0.22 MB DOC)

Text S2 CONSORT checklist.
(0.19 MB DOC)

## Abbreviations

ACASI: audio computer-assisted self-interview
CI: confidence interval
DUA: discordant unprotected anal sex
MSM: men who have sex with men
OR: odds ratio
UA: unprotected anal sex
