# Supplementary material for: An Intervention to Reduce HIV Risk Behavior of Substance-Using Men Who Have Sex with Men: A Two-Group Randomized Trial with a Nonrandomized Third Group
Source: PLoS Med. 2010 Aug 24;7(8):e1000329. doi: 10.1371/journal.pmed.1000329 (PMC2927550; doi:10.1371/journal.pmed.1000329)
Supplement: Text S1 — Trial protocol. (0.22 MB DOC) [file pmed.1000329.s001.doc]

* * * 6-5-2006 * * *

**Protocol #3985**

**Project Mix**

*Behavioral Intervention to Reduce Sexual Risk Behavior of*

*Substance-Using (Non-Injection) Men Who Have Sex with Men*

**Protocol for Trial Phase**

CDC, NCHSTP, Division of HIV/AIDS Prevention, Prevention Research Branch

University of Illinois-Chicago & Howard Brown Health Center

Health Research Association, Los Angeles

New York Blood Center & New York Academy of Medicine

San Francisco Department of Public Health & University of California, San Francisco

Table of Contents

I. Project Overview 4

A. Protocol Summary 4

B. Study Sites 5

C. Investigators/Collaborators 5

II. Introduction 8

A. Background Information 8

B. Study Justification 8

III. Focus Group Phase of the Protocol (previously approved by IRB, September 2003) 11

IV. Pilot Phase of the Protocol (previously approved by IRB, March 2004) 12

V. Trial Phase of the Protocol 13

A. Methods 13

1. Study Design 13

2. Trial Cohort 13

3. Study Population 14

a) Description of study population 14

b) Participant inclusion criteria 15

c) Participant exclusion criteria 15

d) Estimated number of participants 16

4. Non-Randomized Control Arm (3^rd^ Arm) 17

B. Study Procedures 17

1. Participant Recruitment Procedures 17

a) Active recruitment 17

b) Passive recruitment 19

2. Pre-screening 19

3. Full Screening 21

4. Baseline Assessment Individual Session 27

a) Confirmatory Screening 27

b) Written Informed Consent 28

c) ACASI Survey 28

d) HIV Counseling and Testing 29

5. Randomization at First Group Session 32

6. Intervention and Comparison Arm Sessions 34

7. Quality Assurance 36

8. Follow-up 37

9. Retention Efforts 40

C. Logistics and Reimbursement 45

1. Time 45

2. Compensation 45

D. Risks and Benefits 47

1. Risk to Participants 47

2. Confidentiality 48

3. Benefits 48

E. Data Management and Analysis 50

1. Data Management 50

2. Data Analyses 51

a) Qualitative data 51

b) Quantitative data 51

3. Information Security 51

4. Dissemination, Notification, and Reporting of Results 52

a) Notifying participants of individual results and study findings 52

b) Dissemination of findings 53

References 54

Appendices 56

**Project Mix**

*Behavioral Intervention to Reduce Sexual Risk Behavior of*

*Substance-Using (Non-Injection) Men Who Have Sex with Men*

# I. Project Overview

The purpose of this project is to test the efficacy of an HIV prevention behavioral intervention to reduce sexual risk for HIV infection among non-injection, substance-using men who have sex with men (SUMSM). This study is a collaborative project with multi-site research teams funded through a cooperative agreement. The primary goal of the intervention is to reduce HIV transmission by reducing the incidence of unprotected anal sex while under the influence of alcohol and other drugs (AOD). In the focus group component testing phase of the project, we examined the feasibility and acceptability of **five** of six intervention sessions. In the pilot phase of the project, we followed one cohort (per site) through baseline assessment and the full six sessions of the intervention, including a follow-up session to gather feedback. **This protocol describes the trial phase only.**

## A. Protocol Summary

At an earlier date, we requested and received permission to conduct focus groups for component testing the individual two-hour sessions of a multi-session behavioral intervention, specifically testing sessions 1 through 5 of a six-session intervention. The overall purpose of the focus group component testing was (1) to assess feasibility and acceptability of content and flow of session activities, and (2) to gain insight into additional activities (besides those presented in the focus group) that may be useful in reducing sexual risk among substance-using MSM. After experiencing a particular intervention session, participants gave direct feedback about the session in a group discussion fielded by a staff person other than the facilitators who headed the intervention session. Each site conducted 2-9 focus groups that include a target of 8-10 persons per group (minimum 5, maximum 12). The present document includes revisions to the intervention sessions based on feedback and observations gained during the focus group phase.

The pilot phase enrolled one cohort at each study site, including intervention group and comparison group arms. A target of 5-10 men participated in each arm (intervention and comparison groups) of the cohort at each site, totaling 10-20 men per site and 40-80 men across sites.

## B. Study Sites

The focus groups, full pilot test of the intervention, and the intervention trial will be conducted - in conjunction with the CDC - in four sites: Chicago, Los Angeles, New York and San Francisco. Following are project staff and their respective institutions/sites:

## C. Investigators/Collaborators

CDC, NCHSTP, DHAP, Prevention Research Branch

Gordon Mansergh, PhD, Project Officer (oversight of project)

Stephen Flores, PhD, Co-Project Officer (assist with oversight of project)

Pilgrim Spikes, PhD, Project Coordinator (content expertise, administrative support)

Tracie Wright-Schnapp, MPH, Project Coordinator (process expertise, administrative support)

David Purcell, PhD, Consultant (content and process expertise)

Ron Stall, PhD, Consultant (content and process expertise)

Health Research Association (Los Angeles)

Sharon Hudson, PhD, Principal Investigator (site oversight of project)

Peter Kerndt, MD, Co-Principal Investigator (content expertise)

Bobby Gatson, Project Coordinator (content expertise, administrative support)

John Copeland, BA, Community Specialist (content expertise, administrative support)

New York Blood Center & New York Academy of Medicine

Beryl Koblin, PhD, Principal Investigator (site oversight of project)

Victoria Frey, PhD, Co-Principal Investigator (content expertise)

Sebastian Bonner, PhD, Co-Principal Investigator (content expertise)

Jay Loeffel, MEd, Administrator (administrative support)

Kent Curtis, MSW, Project Coordinator (content expertise, administrative support)

Sarah Sisco, Program Manager (content expertise)

Josh Bonelli, MSW, Recruitment and Retention Coordinator (content and process expertise)

University of Illinois – Chicago & Howard Brown Health Center

David McKirnan, PhD, Principal Investigator (site oversight of project)

Lawrence Ouellet, PhD, Co-Investigator (content expertise)

Antonio (Dave) Jimenez, MA, Co-Investigator (content expertise)

Carol Ferro, MPH, Project Coordinator (content expertise, administrative support)

Chris Powers, BA, Coordinator of Recruitment and Retention (content and process expertise)

Anna Veluz, BA, Graduate Assistant (data analysis and process expertise)

PHFE Management Solutions, Inc. & San Francisco Department of Public Health AIDS Office

Grant Colfax, MD, Principal Investigator (site oversight of project)

Greg Greenwood, PhD, Co-Principal Investigator (content expertise)

Tim Matheson, PhD, Project Coordinator (content expertise, administrative support)

Rob Guzman, MPH, Project Coordinator (content expertise, administrative support)

Gavin Morrow-Hall, Recruitment Coordinator (content and process expertise)

Nicole Lightburn, BA, Recruitment Coordinator (content and process expertise)

# II. Introduction

## A. Background Information

Men who have sex with men (MSM) continue to be the largest risk category for incident and prevalent cases of HIV and AIDS in the U.S. (CDC, 2002). Studies of MSM have established an association between AOD use and risky sexual behaviors (Colfax et al., 2001; McKirnan et al., 2001; Purcell et al., 2001; Reback & Grella, 1999; Seage et al., 1998; Stall et al., 2001). AOD use exists in many MSM social contexts (e.g., bars, clubs, circuit parties) and may facilitate sexual risk (Halkitis et al., 2001; Mansergh et al., 2001; Purcell et al., 2001). In fact, Chesney and colleagues (1998) found recent drug use to be associated with recent HIV seroconversion. Although studies have assessed and confirmed the association of AOD use and unsafe sex, few have focused specifically on reducing sexual risk of SUMSM.

In a meta-analysis examining research on sexual risk reduction interventions for MSM (Johnson et al., 2002), nine controlled intervention trials were identified. Overall, they showed significant protective effects indicating that HIV interventions for MSM are generally effective at reducing sexual risk behaviors among MSM. However, none of these interventions specifically targeted non-injecting SUMSM. Thus, a gap exists in prevention efforts to reduce HIV transmission among SUMSM.

## B. Study Justification

The primary aim of the Project Mix overall is to develop and evaluate a practical intervention strategy that is specifically tailored to the needs of non-injecting SUMSM. The intent of the intervention is to decrease the number of unprotected anal sex partners (and acts) while under the influence of AOD and in general. This study is consistent with previously identified research priorities within NCHSTP.

The primary purpose of the **focus group phase was** to test the feasibility and acceptability by session of a pilot behavioral intervention to reduce sexual risk of MSM in the context of AOD use and in general. More specifically, the intent of the focus groups were :

- To observe and gain direct feedback from participants about whether and to what degree the intervention session activities work as intended;
- To assess the amount of time needed for the intervention session activities.

A secondary purpose of the focus group phase was to gather ideas about additional activities besides those presented in the focus groups that could be useful in reducing sexual risk among substance-using MSM.

Based on focus group findings, intervention session activities were revised for testing in the **pilot phase of the project**. The primary purpose of the pilot phase was:

- To observe and gain direct feedback from participants about whether and to what degree the intervention session and comparison group activities work as intended;
- To assess the amount of time needed for the intervention and comparison group session activities.
- To observe the flow of intervention and comparison group activities within and across the six sessions, including homework assignments;
- To observe the flow of procedures and materials, including HIV counseling and testing and ACASI survey assessment at baseline, randomization to groups at the time of Session 1, retention efforts, and data management.

This document presents the **full trial** protocol that includes revisions based on findings in the pilot phase of the study.

**III. Focus Group Phase of the Protocol** (IRB Approval Received September 2003)

**IV. Pilot Phase of the Protocol** (IRB approval received March 2004)

**V. Trial Phase of Project Mix**

**A. Methods**

## 1. Study Design

The purpose of the **study trial** is to test the efficacy of the six-session multi-component intervention arm versus the comparison arm. The intervention outcomes are to decrease frequency of unprotected anal sex in the context of alcohol/other drug (AOD) use and to decrease the number of unprotected anal sex partners. The intervention content is being revised after considering and incorporating information about feasibility and acceptability gained during the focus group phase (CDC IRB approval received on September 8, 2003) and the pilot phase (CDC IRB approval received March 2004). The trial includes screening, baseline assessment, HIV counseling and testing, six group sessions, and 3- 6- and 12-month follow-up assessments. Recruitment for the study trial will last approximately 21 months.

**2. Trial Cohort**

We plan to complete approximately 18-25 group cohorts per site. Each cohort includes one intervention group and one comparison group with 5-10 men (**mean of 7**) randomized into each group. Using active and passive recruitment methods, sites will recruit participants who meet the study eligibility criteria. “Active” recruitment includes outreach recruitment at street and venue locations where SUMSM socialize or congregate, such as bars, clubs, gay businesses, agencies and through websites and voluntarily-provided email address databases or listserves at each site. Sites will “passively” recruit men through flyers and information cards, and through referrals from local agencies and organizations, community advisory board members, and other study participants. Potential participants will be screened and, if eligible and willing to participate, will voluntarily provide written informed consent. A baseline visit will be conducted involving a behavioral assessment, HIV counseling and an HIV antibody test (except for HIV antibody positive men who provide documentation – see *Full Screening* section below). This baseline and testing session is expected to last approximately two and a half hours (range, 2-3 hours). At an assigned time, men in a cohort will return for Session 1 to be randomized to intervention and comparison group arms. Each of the six group intervention and comparison sessions will be held weekly unless extenuating circumstances arise. Each session lasts approximately 2 hours. Group sessions will be held in the following locations: New York Blood Center Project ACHIEVE research site at Union Square, New York City; a field office in Hollywood, California; Howard Brown Health Center and field offices in Chicago; San Francisco Department of Public Health AIDS Office, and a CBO in the East Bay, Oakland, California. For purposes of quality assurance (see section on Quality Assurance), the intervention and comparison group sessions will be audio recorded and observed occasionally by 1-2 staff members for review.

## 3. Study Population

### *a) Description of study population.*

The study population will consist of non-injecting HIV-negative and HIV-positive SUMSM who live in the four metropolitan areas under study (Chicago, Los Angeles, New York, and San Francisco). Being an important and unique characteristic of the sample, the balance of HIV status will be monitored throughout the study, and efforts will be taken to adjust and target recruitment efforts so that one serostatus does not exceed 60% of the sample. Each site will either recruit an ethnically diverse sample or conduct the group in a largely Latino or African-American geographic catchment area (to be determined and balanced across the four sites). For the study overall, sites will over-sample Latino and African American men for adequate representation due to high HIV prevalence rates among these populations. Similar to HIV status, race/ethnicity will be monitored throughout the study and recruitment will be adjusted and targeted with the intent of having relatively balanced proportions of Latino, Africa-American and white participants in the sample (approximately 30% for each group) and no more than 50% for any one race/ethnicity.

### *b) Participant inclusion criteria.*

To participate in the trial, the men must:

i. be 18 years of age or older;

ii. self-identify as male;

iii. understand and read English;

iv. live within the metropolitan area;

v. report being drunk or “buzzed” on alcohol 2 or more times or high on non-injection drugs at least once during (or two hours before) anal sex in the past six months;

vi. have had at least one unprotected anal sex episode in the past six months with a male partner of unknown or different HIV-status than their own (i.e., DUA)

### *c) Participant exclusion criteria.*

Men are ineligible to participate in the trial if they:

i. report only marijuana, only Viagra or only marijuana and Viagra use during anal sex (current research shows that marijuana use is not associated with sexual risk behavior);

ii. report injecting drugs (eligible if only methamphetamine, steroids, hormones and/or prescribed mediations) in the past six months;

iii. have known their HIV-positive status for less than six months (this is done to allow for psychological and behavioral adjustment following an initial HIV diagnosis and this time frame coincides with our screening window); these men (if they so desire) will be put on a wait list and contacted after 6 months have passed since their first HIV-positive test result;

iv. are currently involved in another HIV behavioral intervention study for MSM [local study site identifies a list of such studies in their community at the time of the trial];

v. have a specific plan to move from the metropolitan area within the next 15 months;

vi. participated in the pilot phase of the project;

vii. other reasons that the investigators deem would make participation either detrimental to the participant or to the study.

### *d) Estimated number of participants.*

The trial will be conducted to test the efficacy of a six-session behavioral intervention curricula. Given 30% attrition between baseline and randomization and 15% attrition between randomization and 12-month follow-up (see Table 3), a total of 1428-2000 men (357-500 per site) are needed at baseline in order to get at least 1000-1400 men at randomization (250-350 per site) and 852-1192 at 12-month follow-up (213-298 per site). Thus for a mean of 7 (minimum 5, maximum 10) men per group for each arm of a cohort, 18-25 group cohorts will be conducted at each site with a new cohort beginning approximately every 4-6 weeks (i.e., the week following completion of a cohort). Participants of the earlier pilot phase are not eligible to participate in the trial, however participants in the focus group phase are eligible for the trial.

**Table 3. Estimated number of participants in the trial to reach n=250-350 randomized in Session 1 and n=213-298 at 12-month follow-up per site.**

Baseline Randomization (Session 1)^a^ Follow-up (12-month)^b^

n n *(18-25 cohorts x 7 [mean] per group x 2 arms)* n

----------------------------------------------------------------------------------------------------------------------------------------

Chicago 357-500 250-350 213-298

Los Angeles 357-500 250-350 213-298

New York City 357-500 250-350 213-298

San Francisco 357-500 250-350 213-298

**Total 1428-2000 1000-1400 852-1192**

----------------------------------------------------------------------------------------------------------------------------------------

^a^70% of baseline (30% attrition from baseline to randomization [Session 1])

^b^85% of randomization (15% attrition from randomization [Session 1] to 12-month follow-up)

**4. Non-Randomized, No-Attention Control Arm (3^rd^ Arm)**

Following completion of the final randomized cohort as outlined in section 2 above, 480 additional men (approximately 120 per site) will be enrolled in a non-randomized control arm of the study. These men will be recruited through similar strategies as the randomized men, required to meet the same eligibility criteria, and complete a full baseline assessment as described. They will, however, receive a different description of the study (i.e., two assessment appointments over 4.5 months) and a coinciding consent form (see Consent Form appendix). Third arm participants will receive a “3-month” follow-up assessment, however no subsequent follow-up appointments due to limited time and funds. At follow-up, we anticipate 85% retention (n=408, or approximately 102 per site), and with 80% power we could detect a change difference of approximately 15% or greater given the anticipated prevalence of our key outcomes for the control arm compared to either of the two randomized arms [see power analysis section]).

The 3^rd^ arm men will not attend any group sessions or other appointments with study staff between the baseline and “3-month” assessment appointments. To account for similar follow-up time periods, the “3-month” follow-up for the Control Arm will actually occur 4.5 months after their baseline appointment (i.e., 3 months plus 6 weeks) to account for the 6 weeks of group sessions for each of the two randomized arms. Similar retention efforts will be employed for the Control Arm as for the other two arms both prior to the baseline appointment and prior to “3-month” follow-up. Participants will receive similar stipend amounts as the other two arms for their baseline and follow-up appointments.

The purpose of the third arm is to provide a no-attention control comparison as secondary to the primary comparison of the randomized attention-control comparison. A randomized control arm was not possible from the beginning of the study due to time and finding, so we propose to do a non-randomized control arm following completion of the randomized cohort arms. It is acknowledged that this approach is not ideal, however it offers scientific merit beyond the 2-arm design in that it provides a comparison arm void of the experience or convening and talking with a group of peers for 6 consecutive weeks. If the 3^rd^ arm does not differ on baseline demographic or key outcome variables with the two randomized arms, then less concern for arm inequality or historical cohort differences will exist. However, if differences do occur in baseline variables, then their influence may be decreased by controlling for them in analysis. In either scenario, non-randomization has important limitations that will be discussed in-depth in subsequent papers and presentations, however it still provides an opportunity to further the science on behavioral interventions that rarely offer 3^rd^ arm comparisons.

**B. Study Procedures**

## 1. Participant Recruitment Procedures

Recruitment of participants for the trial will occur through “active” (i.e., venue outreach, center registry) and “passive” (i.e., referral, flyers/cards) methods. A complete flowchart of recruitment and screening is presented in Appendix DD.

Based on information from key informants, community advisory board members, and previous experience, each site will identify relevant venues, agencies and organizations for recruitment of SUMSM. Recruitment will occur in various geographical catchment areas based largely on race/ethnicity (Latino, African American, white) or in ethnically-diverse locations, including locations frequented by gay and non-gay identified SUMSM.

### *a) Active recruitment.*

Each man approached will be given a project information card or flyer that provides a telephone number and basic information regarding the study (Appendix EE). Potential participants will be given information about the general nature of the study trial, the time involved (number of appointments and approximate length of time: one 2.5-hour behavioral assessment session, six 2-hour group sessions, and three 2-hour follow-up sessions), and informed that the reimbursement is:

1. $40 for the baseline individual session for SF and Chicago ($25 for NY; $35 for LA) (see Appendix FF for scripts);
2. $25 for each of the 6 intervention sessions in NY and Chicago ($35 in LA and SF); and
3. $35, $40 and $50, respectively, for 3, 6, and 12-month follow-up assessment visits in all sites except NY where it will $25, $35 and $50 respectively.

If possible, recruiters will collect limited contact information (e.g., first and last name, phone number, email address) in order to contact and screen potential participants on the phone; screening will **not** be conducted by email. Interested potential participants will also be asked if a phone message may be left on an answering machine, what the **best and preferred** way and time to contact them is, and a street address for mail contact. If contact information is not collected from a potential participant at the recruitment venue, a study information card (including project phone number) will be given to the man so he can call the office for screening. Each site will maintain their own list of potential participants under strict confidentiality and locked files, and attempt to contact those men for screening. A central list of focus group, pilot and trial participants at each site will be securely stored in a locked cabinet through completion of recruitment for the intervention trial (expected date: Summer 2006), at which point the list will be destroyed. A central list is kept in order to verify that focus group participants are not involved in the pilot phase and that pilot participants are not involved in the trial, and to allow for focus group participants to participate in the intervention trial. Active recruitment consists of street outreach recruitment at street and venue locations, including bars, clubs, circuit parties, festivals, gyms, gay businesses, sex clubs, parks, other public sex environments, gay websites (e.g., M4M4sex.com, manhunt.com, craigslist.org), voluntarily-provided email address databases from each site, and other locations where SUMSM socialize or congregate.

### *b) Passive recruitment.*

##### i. Flyers, information cards and advertisements. Passive recruitment will involve posting trial recruitment flyers and placing project information cards in locations accessed by gay and non-gay identified SUMSM (e.g., gyms, bars, clubs). Advertisements in local gay or other newspapers and magazines may be used if it is difficult recruiting participants by other methods. [Drafts of the flyers are attached (see Appendix EE)]

ii. Referrals and word-of-mouth. Referrals may be made through local agencies and organizations that provide programs and services for MSM (e.g., AIDS service organizations, health clinics), project community advisory board members, and previous study participants. Agencies and community advisory board members will be instructed to use the language included in the information cards and flyers as a guide when describing the study to potential referrals. They will be asked to encourage potential participants to call the study phone number if they have further questions about the study. Pilot recruitment flyers and information cards will be placed at these locations as well.

## 2. Pre-Screening

During active recruitment outreach to street and venue locations, men may be pre-screened using Personal Digital Assistants (PDAs) or “Palms” using a brief tally of 7 broad eligibility criteria (see Appendix GG); **pre-screening** may be conducted by email (except in Chicago) because of its less sensitive nature than screening (see below). The recruiter will ask the participant the questions verbally and record their responses on the PDA. If eligible on the prescreener and willing to be screened, the recruiter will ask if it is okay to get their name, phone number, and email address to contact them for full screening. If given, contact information is written on a contact information sheet, which is visually separate from data in the PDA. This is done intentionally to avoid even a perception of linked or linkable data with personal identifiers within the PDA. The same procedure is followed at each site.

Prescreening is done to reach as many men as possible and to minimize the amount of staff time and effort needed for full screening. In pre-screening, brevity is key. Because less sensitive items are asked during pre-screening (e.g., illegal drug use alone is not asked [alcohol and drug use are combined], unprotected anal sex is not asked), informed consent is not proposed. **Brief verbal informed consent will be obtained for prescreening and full screening (see next section). Full written consent will also be obtained for trial participation (see below), as during the focus group and pilot phases which received prior approval from IRB (September 2003 and March 2004, respectively).** The following verbal script will be used to describe the pre-screening process, as outlined in Appendix FF:

"Hi. I work with [e.g., SFDPH, HRA, HBHC or NYBC] on a research study called Project Mix to learn more about health behavior of men who have sex with men. Can I ask you a few questions? Some of the items are personal, and it only takes a minute or so. [If an affirmative response is given…]

- Are you 18 or older?

- Are you a resident of the [Chicago/Los Angeles/New York/San Francisco Bay] area? If yes, do you have a specific plan to leave the area within the next 15 months?

Now, the next few items are more personal…

- Have you used alcohol or other drugs in the past 6 months?

- Have you had anal sex with a man in the past 6 months?

- Have you had **oral** sex while drunk or high in the past 6 months?

- Have you had **anal** sex while drunk or high in the past 6 months?

- What do you consider to be your primary race/ethnicity? (*note: this is not an eligibility criterion but a venue targeting criterion, and thus important information to gather for targeted recruitment)*

We request that informed consent be altered and some elements be waived for **prescreening and screening only** (note that trial enrollment will follow full written informed consent procedures [**see the following section titled “Written Informed Consent Process for Trial Participation”**]) given the following criteria stated in 45 CFR 46.116(d):

a) The research [i.e., prescreening] involves no more than minimal risk to the subjects;

b) The waiver or alteration will not adversely affect the rights and welfare of the subjects;

c) The research could not practicably be carried out without the waiver or alteration; and

d) Whenever appropriate, the participants will be provided with additional pertinent information after participation [i.e., being prescreened].

We also request waiving documentation of informed consent for **prescreening and screening only**, which may be obtained if “the research presents no more harm than minimal risk of harm to subjects and involves no procedures for which written consent is normally required outside of the research context” [45 CFR 46.117(c)(2)]. Following is justification for these requests specifically for **prescreening** (justification specifically for **screening** is presented in the next section).

Men will have voluntarily agreed to participate in the **prescreener**. The prescreener takes only a minute or so, and the participant has the direct opportunity to refuse to participate in the full screening or to give personal contact information if eligible on the prescreener; the participant can also decline participation at any point during the prescreener, screener, or the study. Prescreening is not easily completed if staff must verbally and/or in written form cover all required criteria of informed consent prior to screening. Such a consent process requires much more time than prescreening itself, and potential respondents could grow weary and discontinue the conversation, especially when prescreening on the street. After prescreening is completed and participants are eligible and willing to be screened, they will be told more about the subsequent screening process and the study overall, including information about time involved and compensation for their time if they are eligible on the screener.

If a man is eligible on the pre-screening criteria, he will be asked if he is willing to participate in a full screening at a later time. The recruiter will first ask if the person is willing to provide minimal contact information (name, phone number, email address) so that a staff person could call them for screening at a later date. Interested potential participants will also be asked if a phone message may be left on an answering machine, what the best way and time to contact them is, and possibly a street address for mail contact. If the person declines to provide contact information, the recruiter will give the potential participant a project information card and request that he call the project office directly for screening. Men who provide contact information will also be given a project information card and told that they may call the project office if they desire further information about the study. Full screening will not be conducted via email so as not to electronically transfer potentially sensitive information about a person associated with their email address. However, pre-screening may take place via email because of the less sensitive nature of the information being gathered (e.g., no exclusive questions about illegal drug use [alcohol is included in the substance use items] or unprotected anal sex). **[Chicago will drop the last part about pre-screening by email].**

## 3. Full Screening (Screener 1)

Potential participants will either call the project phone number or, if contact information is gathered at the recruitment venue, be called or emailed by project staff. Screening will be conducted over the phone or in person for walk-ins; screening will **not** be conducted by email. Each potential participant will be screened for eligibility using the full screening instrument (Appendix HH), and the following verbal script is a guide to describe the screening process and privacy issues, as outlined in Appendix FF:

1. Randomized Arms:

"Hi. I work with [e.g., SFDPH, HRA, HBHC or NYBC] on a research study called Project Mix to learn more about health behavior of men who have sex with men and to work to protect their overall sexual health. The study will involve (number of appointments and approximate length of time) one initial individual 2.5-hour appointment and six 2-hour group sessions over the next two months, and then a 2-hour appointment at 3-, 6-, and 12-months thereafter. To see if you are eligible, I need to ask you a few questions. It will only take a minute or two. Some of the questions are personal. Your involvement is voluntary, your responses will be kept private, and you can refuse to answer a question or stop at any time. Information we gather from you will not identify you individually. Are you interested?”

Once interested participants complete the screening procedure as eligible for study participation and express interest in attending, the reimbursement will be described as follows (note that if participants ask about this information at any point during the full screening process, this information will also be given):

“If you decide to participate in the study, you will receive $40 ($25 in NY; $35 in LA) for an initial individual 2.5-hour appointment, $25 ($35 in Los Angeles and San Francisco) for participating in each of six 2-hour group sessions, and $35, $40 ($25, $35 in NY) and $50 for participating in each of the three 2-hour follow-up visits, at 3, 6 and 12-months, respectively. That is a total of seven sessions in the next two months and three follow-up visits in the year after that."

1. Non-randomized 3^rd^ Arm (recruited after the 2 randomized arms are completed):

"Hi. I work with [e.g., SFDPH, HRA, HBHC or NYBC] on a research study called Project Mix to learn more about health behavior of men who have sex with men and to work to protect their overall sexual health. The study will involve one initial individual 2.5-hour appointment and a 2-hour appointment 4 and a half months later. To see if you are eligible, I need to ask you a few questions. It will only take a minute or two. Some of the questions are personal. Your involvement is voluntary, your responses will be kept private, and you can refuse to answer a question or stop at any time. Information we gather from you will not identify you individually. Are you interested?”

If the person is interested:

“If you decide to participate in the study, you will receive $40 ($25 in NY; $35 in LA) for an initial individual 2.5-hour appointment, and $35 ($25 in NY) the 2-hour follow-up visit 4 and a half months later. That is a total of two sessions within the next five months."

We request that informed consent be altered and some elements be waived for **pre screening and screening only** (note that trial enrollment will follow full written informed consent procedures [**see the following section titled “Written Informed Consent Process for Trial Participation”**]) given the following criteria stated in 45 CFR 46.116(d):

a) The research [i.e., screening] involves no more than minimal risk to the subjects;

b) The waiver or alteration will not adversely affect the rights and welfare of the subjects;

c) The research could not practicably be carried out without the waiver or alteration; and

d) Whenever appropriate, the participants will be provided with additional pertinent information after participation [i.e., being screened].

We also request waiving documentation of informed consent for **prescreening and screening only**, which may be obtained if “the research presents no more harm than minimal risk of harm to subjects and involves no procedures for which written consent is normally required outside of the research context” [45 CFR 46.117(c)(2)]. Following is justification for these requests specifically regarding **screening** (justification specifically for **prescreening** is presented in the prior section).

Men will have voluntarily called the researchers to screen for study participation after seeing flyers, hearing about the study by word-of-mouth, or voluntarily giving their contact information to staff during recruitment outreach. The full screener takes only a few minutes, and the participant has the direct opportunity to refuse to participate in the study immediately after screening; the participant can also decline participation at any point during the screener or the study. Contact information collected will be destroyed if individuals decline to participate during or after the screening process. Passive declines are respected in that after a maximum of **three** attempts to contact a person for screening with no replies from the potential participant, the person will be withdrawn from the active list and their contact information destroyed. If (non-decline) responses are received from the potential participant, then a count of three attempts to contact may begin again after that point. If a response to decline participation is received from the potential participant at any point, staff will not attempt to contact that person further for screening. Screening is not easily completed if staff must verbally and/or in written form cover all required criteria of informed consent prior to screening. Such a consent process requires much more time than screening itself, and potential respondents could grow weary and discontinue the conversation, especially when screening over the phone. After screening is completed and participants are eligible, they will be told more about the process they will encounter when they arrive for their scheduled baseline individual session, including information about written informed consent, time involved and compensation for their time (see the next paragraph).

The full screening process also covers the following information about the trial and compensation for time. This information is given at screening so that disclosure of important study procedures is provided at the point in time a person agrees to participate in the trial. Men who are eligible and agree to participate in the trial are told:

- Participants will be re-screened when they arrive for the baseline behavioral assessment session.

- Participants who arrive at the baseline behavioral assessment session too late to complete the written consent and assessment process in the time allotted, depending on staff availability, will not receive compensation and, if possible, they will be rescheduled for another time;

- Participants who arrive too late to be (randomly) assigned to a group at their first group session (approximately 15-30 minutes after the scheduled appointment time [but before the session content starts, i.e., during introductions and group rules]) will not receive compensation and will not be eligible for that cohort;

- If less than twelve participants show up at the first group session, the men who showed up will be reimbursed $15 for their time and will be rescheduled for another time if possible;

- If more than 20 participants (10 per group) show up for the first group session, those who arrived after the first 20 men will not be assigned to a group and will receive $15 for their time and will be rescheduled for another time if possible;

- Men who self-report an HIV-positive serostatus at screening are asked to provide appropriate documentation indicating that they are HIV positive at or before the baseline behavioral assessment session. Appropriate documentation includes government-issue photo identification and at least one of the following: an HIV medicine prescription bottle with name on it; a letter from physician, provider, or agency (including a case manager) that states participant’s name and positive HIV status; AIDS Drug Assistance Program [ADAP] documentation; a positive test result with participant name (this can also be faxed or delivered to the local staff offices [fax number and address will be provided at screening] within 7 days of screening or brought to the baseline session). If appropriate documentation is not received by the baseline appointment, HIV-positive men will be tested using the OraSure oral fluid test to confirm their self-reported HIV-positive status (see the HIV Counseling and Testing section below for the full procedure). Participants who self-report HIV positive status, regardless of documentation provided, will receive counseling equivalent to post-test counseling received by other study participants. This counseling will include personalized exploration of risk, developing a risk-reduction plan, as well as referrals in one brief session (Also see *HIV Counseling and Testing* below).

- Participants who arrive more than 30 minutes late to sessions 2 through 6 will be encouraged to participate in the group, but will not receive reimbursement for that session.

Recruiters will attempt to contact potential participants up to three times if they do not hear back from the person. If no contact is made within two weeks after leaving the third message, that person is considered a passive decline to participate. When making phone calls, staff will only talk to the participant directly unless the participant has authorized us to leave a message. If the participant is not at the contact number and someone else answers the telephone, staff will leave a general message (e.g., say they are from Project Mix, and leave their first name and telephone number). If contact is not made with the first call and participants have given permission to leave a message on their answering machines, a generic message will be left to return the call (see Appendix II for script). If the participant does not reply within a few days or a week, another attempt to contact will be made. A similar generic message will be left. If the potential participant does not reply after the second attempted contact, a third and final attempt will be made. If a reply is not received within two weeks following of a third attempt, a passive decline is assumed. Three attempts at contacting a potential participant may commence each time after a (non-decline) response (e.g., phone or email message) is received by a potential participant. If a message is left by the participant declining participation, the person will not be contacted further for screening.

Contact information will be destroyed if men decline involvement in the study, however a tally of ineligibles and declines (active and passive) will be maintained. Contact information for trial participants will be kept in a central, locked file and destroyed six months after the study is completed **unless** participants indicate to staff (see Locator Form, Appendix JJ) that:

(a) staff may keep their contact information for potential participation in future studies or programs, or

(b) they want to receive a summary of primary results following completion of the study. Additionally, all contact information will be de-linked from study IDs for all participants six months after the study is completed. Trial participants will be informed of this procedure. This step is taken for purposes of follow-up assessment and to prevent pilot participants from participating in the full intervention trial. However, focus group participants are allowed to participate in the trial.

Secured information about men who participated in the pilot phase will be referenced during trial enrollment in order to verify that they indeed did not participate in the pilot. If it is determined during trial enrollment that a man participated in the pilot, they will be deemed ineligible to be in the trial and cordially told so. They may be referred to other local studies as appropriate.

**4. Baseline Assessment Individual Session**

*a) Confirmatory Screening.*

Upon arrival at the enrollment visit and prior to written informed consent procedures (described below), all individuals will be re-screened for eligibility in a semi-private location using the same instrument described in *Full Screening* above. This procedure is done to confirm that the participant walking in is the same as the participant screened and scheduled on the phone, and to re-assure eligibility for participation in the study. They will provide verbal informed consent to participate in a brief screening interview also as described above. Individuals who screen eligible will then complete written informed consent procedures. Those who do not screen eligible will be informed, reimbursed $15 and thanked for their time just as with the full screening procedure (see Appendix FF).

*b) Written Informed Consent Process for Trial Participation*

We will be using ***written informed consent procedures for enrolling men into the study trial***. During the baseline individual session, participants will read or have summarized to them a voluntary informed consent form (Appendices KK through NN by site). The informed consent form provides details of the study procedures, risks, benefits, site contact information, and the nature of confidentiality and voluntary participation. The consent process also covers information on the trial and compensation for time as outlined in the Screening section above.

Before a participant signs the informed consent form, staff will review the form, ask if the participant understands the content of the consent form, and answer any questions they may have. Participants will be given a copy of the informed consent form for their records.

*c) ACASI Survey.*

After successfully completing written consenting procedures, the participant will complete the baseline assessment on a computer in a semi-private location. The baseline assessment session of the trial consists of a 1-hour (approximately) Audio-Computer-Assisted-Self-Interview (ACASI)-administered survey of self-reported demographic factors, recent sexual and drug use behavior, and a number of potential cofactors of sexual and drug use behavior, including attitudes, beliefs, knowledge, traits and other psychosocial factors (see Appendix OO for the paper version of the ACASI baseline survey instrument). Staff will be present to assist the participant in getting started on the ACASI computer, which includes a set of practice items. Staff will remain easily accessible during survey completion to address any technical problems or to answer questions participants may have. Staff may periodically check in with the participant to inquire about any difficulties that may arise in completing the survey. However, to ensure privacy, the staff person will not directly observe the full process of completing the survey on the ACASI. Then staff will deliver HIV counseling and testing in a private location (see Appendix PP for baseline session flow).

*d) HIV Counseling and Testing.*

Following completion of the ACASI survey, the participant will receive counseling and complete rapid HIV testing (OraQuick or Rapid OraSure [if available at the time]) if they report receiving a negative result following their most recent HIV test or they self-report that they have not been tested or do not know their HIV-status. These participants will have the OraQuick finger-stick rapid HIV test (or Rapid OraSure oral swab HIV test, if available) administered consistent with instructions included in the test packaging (see Appendix QQ attachment: OraQuick Guide.pdf). These tests are CLIA-waived and include 3 steps before reading a result: (1) collecting a sample from a finger stick or oral swab using the specimen collection loop; (2) mixing the sample by inserting the loop into the developer vial; (3) testing the sample by inserting the test device into the developer vial and using a timer, allowing 20 minutes to pass (but no more than 40 minutes) before reading the result.

The oral swab HIV tests involve placing a specially treated swab between the lower cheek and gum for between 2 to 5 minutes. This procedure draws oral fluid onto the swab which is then placed in a developer tube and sent away to a lab to read the results. If the rapid oral test is available for use at the site, results will be given during the counseling visit. An initial reactive test is followed up with a regular OraSure test (if available and the initial test was a rapid HIV test), or a Western Blot test to confirm the presence of HIV antibodies.

Participants whose result is negative will be notified of their negative test result and will receive post-test counseling (described below). They will then be scheduled for a randomization appointment. Participants whose result is positive will be informed of their preliminary positive test result and will receive post-test counseling. These preliminary positive participants will have an oral fluid sample collected using an OraSure oral fluid HIV test kit or have a blood draw performed. The result of a Western Blot test performed on this sample will serve as confirmation of their HIV status. Participants will be scheduled to return for the result of this test approximately 5-10 days after the enrollment visit (depending on local lab schedule). If this confirmatory test is positive, participants will be –if they wish – referred to appropriate medical services and their name will be placed on a 6-month waitlist for participation later in the trial. If this test result is negative, the participant will be referred to have blood drawn in order to perform a Western Blot confirmatory test. If this confirmatory test is negative they will be eligible to enroll in the study and be scheduled for a randomization appointment. If this test result is positive they will be waitlisted.

Participants’ newly diagnosed HIV-positive status is reportable in two study sites (Los Angeles and San Francisco) and exempt from reporting in the other two sites (Chicago and New York City). California state law requires reporting of HIV-positive status using a non-name code. A code is developed based on four elements (a Soundex system: a code based on last name, an 8-digit date of birth, gender (Female, Male, Female-to-Male, Male-to-Female), and the last four digits of the individual’s social security number). Use of this non-name code protects individuals’ privacy in a manner consistent with current California Health and Safety code (H&S 120975-121035). In Illinois, according to state code (77 Illinois Administrative Code 693, see §693.20, ‘Reportable STDs and Laboratory Results’) HIV reporting is not required for the proposed study due to the following: Participants will be participating in an IRB-approved research study which is not primarily intended to provide medical treatment and all personal identifiers are removed from the specimen before testing. In New York, consistent with Public Health Law §2786, HIV-positive test results are similarly exempt from reporting in this study.

All participants who self-report HIV-negative or unknown status will receive pre-test and post-test counseling in conjunction with the rapid test procedures. The counseling protocol is based on CDC’s RESPECT-2 (http://www.cdc.gov/hiv/projects/respect-2). This protocol was designed for use with the rapid test and involves about a 20-30 minute pre and post HIV test counseling session. This counseling involves an orientation to the rapid testing procedure, a personalized exploration of risk, developing a risk-reduction plan, understanding the test result, and identifying sources for support and referrals. The content of this counseling is consistent with CDC recommendations for HIV testing and counseling (CDC, 2001).

Men who report being HIV-positive will not require testing if they produce adequate documentation of their HIV-positive status (i.e, government-issue photo identification and at least one of the following in the participant’s name: an HIV medicine prescription bottle; a letter from physician, provider, or agency [including a case manager] that states participant’s positive HIV status; a positive test result; a lab report with viral load values that indicate HIV positive status; AIDS Drug Assistance Program [ADAP] documentation). If self-reported HIV-positive men do not send or deliver appropriate documentation of their HIV-positive status prior to, or bring with them to, the baseline behavioral assessment, they will be given the option of (1) rescheduling or (2) being tested for HIV during that visit using the OraSure oral fluid test to confirm their self-reported HIV-positive status. A Western Blot will be performed on these samples. In the unlikely event that these test results are discrepant (i.e., negative or indeterminate), participants will be scheduled for a follow-up visit in order to perform a blood draw to confirm their HIV status.

Participants who self-report HIV positive status, regardless of documentation provided, will receive counseling equivalent to post-test counseling received by other study participants. This modified counseling will include comparable content to that received by participants who self-report negative or unknown HIV status. Also based on CDC’s Project RESPECT-2 (http://www.cdc.gov/hiv/projects/respect-2), it involves one [brief twenty-five-minute] session. This counseling involves a personalized exploration of risk, developing a risk-reduction plan, and identifying sources for support and referrals. The content of this counseling is also consistent with CDC recommendations for HIV testing and counseling (CDC, 2001).

Requirements for training to use the rapid test vary across states and counties. New York State, California and San Francisco County each have their own training for the rapid test. Staff at each site have either completed or are in the process of completing this training. Illinois has not defined any requirements so far for training to perform the rapid test. Our study site in Chicago therefore plans to attend a local CDC-sponsored three-day training and receive training from a representative of OraSure laboratories on using the rapid test.

Baseline assessment information is good for 3 months, after which a participant needs to complete the entire baseline appointment again using the same ID. Expired baseline data will be overwritten by new data, and appropriate notation reflecting this will be made in the participant tracking database record. A participant may not complete the baseline more than two times if the reason for the second baseline is due to the participant not showing up or not arriving on time at their confirmed randomization session because of reasons under their control (staff judgment).

**5. Randomization at First Group Session (Session 1)**

When participants arrive at the appointed time for their first group session (Session 1), they will be randomly assigned to either the intervention group or the comparison group. At least 12 people must attend the first session for the cohort to begin (average of 5 per group - allowing 10 to be randomized will result in one group only having 4 participants much of the time). If less than 12 people attend, the cohort will be rescheduled.

Block randomization by HIV status will be generated by an established computer-based program on a staff computer (laptop or desktop) after all participants check in. The computer will generate a list of randomized assignments to the two arms (intervention and comparison) for all men who have checked in by the designated time.

Based on experience with previous studies and the focus group and pilot phases of this project, we expect high risk HIV-negative men to be harder to recruit than high risk HIV-positive men. Thus we plan to use a 4-person block design (2 in Group A, 2 in Group B) to attempt to balance HIV-status between groups in each cohort (Table 4). Because we expect that it will be easier to enroll HIV-positive men into the trial, we plan to use randomly-generated 4 and 6 person blocks to more closely approximate pure randomization, however the first block will be a block of 4 to parallel the 4-block design for HIV-negative men to ensure at least 2 men from each serostatus in each cohort arm.

Every effort will be made ahead of time to schedule for the randomization session (Session 1) at least 4 men of each HIV status. **This is done to minimize the probability of one man of either serostatus being alone in one of the arms.** The exception to this rule if only 1 or 3 persons of a particular serostatus show up for Session 1; it is also possible that if only two people of a particular serostatus arrive at Session 1, they could be split and end up alone in each of the two arms with a 4-block design, in which case he will be randomly assigned to a group in the same process as the rest of the cohort is assigned. Following the group assignment procedure, the computer will randomly assign the two groups (A and B) to type of arm (intervention or comparison) so that group assignment process is blinded up to the final step.

The study coordinator will supervise the randomization process. At the time of the visit, staff will confirm identity, study ID number and that consent was obtained. Once the participants are checked in, the computer will randomly assign the participants to an arm, which will be recorded by staff in the locator database.

A participant may miss up to two confirmed randomization appointments due to circumstances under their control before being dropped (staff judgment).

**Table 4. Plan for block randomization by HIV status: 4-block assignment for HIV-negative men and initial 4-block assignment followed by randomly generated 4- and 6-person block assignment for HIV-positive men (read down columns).**

**HIV+ HIV-**

Cohort1(n=9) Cohort1(n=5)

A A

A B

B A

B B

--- ---

A B

B

A

B

A

Cohort2(n=9) Cohort2(n=8)

B A

A B

A B

B A

--- ---

A B

A A

A A

B B

B ---

Cohort3(n=8) Cohort3(n=6)

A B

B A

B B

A A

--- ---

B A

B A

A

A

---

## 6. Intervention and Comparison Arm Sessions

In addition to **one** baseline assessment session, both randomized arms of the study – intervention and comparison – consist of **six** 2-hour group sessions led by two facilitators. Each participant will be asked to attend **six** session appointments during the first two months followed by **three** 2-hour follow-up assessment sessions, at 3, 6 and 12 months after their final group session. Thus, each participant will attend a **total of ten appointments** during the length of the study, over a 14-month span of time. Two trained and experienced (defined as having experience counseling and facilitating groups and having experience working with populations targeted in this study, i.e., both HIV-positive and -negative SUMSM from a variety of racial and ethnic backgrounds) facilitators will present the intervention or comparison material and activities for the full six group intervention sessions. One or two staff may periodically observe the group sessions and take notes for facilitator and content quality assurance purposes. Content of the intervention arm by session is presented in Appendix RR. Content of the comparison arm by segment (there are twelve 1-hour segments; two segments per group session) is presented in Appendix SS. In both study arms, a resource table will be used at the back of the room in which the sessions are being held. This table will contain printed materials and is intended to provide additional information to participants that desire additional information or resources on certain topics. In the intervention arm, this will include information regarding sexual risk reduction, alcohol and drug treatment programs, as well as mental health services. In the comparison arm, this will include information regarding topics covered in the videos (e.g., local community resources regarding adoption services, spirituality, ethnic and racial community organizations etc.).

At the end of the first group session, the date and time of the subsequent group sessions will be determined. It is expected that group sessions will be held once a week on consecutive weeks, preferably at the same time and day of the week. If holidays interfere, alternative dates will be established.

Participants will be encouraged to attend every session. If a participant misses a session, they will be allowed to attend subsequent sessions, although missed sessions will be documented. If a participant is late for a session, they will be allowed to participate in that session and the extent of their lateness will be recorded in their file in order for researchers to later assess the extent of the content missed (for intervention exposure purposes); participants who arrive late will receive reimbursement in accordance with rules explained to them and described elsewhere in this document. Facilitators will not take significant time away from the group to bring people up to speed on missed content.

**7. Quality Assurance**

As described earlier, each intervention and comparison group at the time of randomization will have a minimum of 5 and a maximum of 10 participants (average, 7). The groups will be audio-recorded and transcribed as needed, and periodically observed, for purposes of facilitator supervision and content quality assurance. The primary purpose of taping the group sessions is to provide an additional and more objective source of assessment information for monitoring and evaluating the quality of the intervention and to be certain the curricula are being implemented as intended. Further analysis (e.g., content analysis) of the tapes may be undertaken to better understand behavior and motivations of study participants and how the study materials may work to facilitate change. As part of the informed consent process, participants will be told about the role of the observers, audio taping, and transcription.

Observers or reviewers of tapes will periodically sit in and take notes during the sessions (approximately 10% of the sessions), and observers or reviewers of tapes will write a brief summary report after they sit in on a group session (see Appendices TT for Session Observer Summary Form). This is done for purposes of both quality assurance (across sites) and facilitator supervision (within sites) to examine the content and process of the sessions for purposes of facilitator supervision and to be sure the curricula are being implemented as intended. Feedback regarding session activity will be discussed among appropriate project staff to ensure adequate and appropriate delivery of intervention content and process; supplemental training of facilitators will be given as needed. Quality assurance across sites consists of two sites (including CDC) reviewing one tape from each cohort arm of each site. Copies of tapes are transferred between sites by FedEx or other courier service, and returned to the original site after reviews are completed. Discussion of the supervisory issues and quality assurance (QA) reviews take place during biweekly QA committee conference calls.

Assessment and counseling staff will observe ACASI and HIV counseling and testing procedures and take note of any problems that arise throughout the trial. Staff will also ask participants if they had any problems with ACASI immediately following completion of the survey. Staff will hold internal reviews and discussions about ACASI, HIV counseling/testing, and clinical/lab procedures during the trial so that recommendations for assessment and procedural improvements can be made on an ongoing basis.

**8. Follow-Up**

Participants will be scheduled for **three** follow-up visits, at 3, 6, and 12 months after their last session. All participants will complete an ACASI behavioral assessment regarding sex and drug risk behaviors and related psychosocial factors (Appendix OOb); this is a reduced version of the Baseline assessment with a few additional items about pre-exposure prophylaxis and methamphetamine use. In addition, participants who were HIV-negative at last testing will provide finger stick blood or oral swab samples for rapid HIV testing at the 12 month visit. Participants will be reimbursed $35 ($25 in NY) for the 3-month follow-up visit, $40 ($35 in NY) for the 6-month follow-up visit, and $50 for the 12-month follow-up visit (to maximize return at the final outcome point of the study, which comes a full 6 months after the prior follow-up visit). Participants who are tested for HIV infection (i.e., men who were HIV negative at last testing) will be given results of their rapid HIV test following procedures described earlier for the baseline assessment. Timing of follow-up visits is scheduled to allow site flexibility while still scheduling follow-up visits within a reasonable time span (see Figure 1).

Every effort will be made to ensure that follow-up visits are not conducted by staff who served as group facilitators (intervention or comparison arm) for that particular participant. This is done to avoid potential reporting bias that may occur when participants interact during assessment with facilitators with whom they worked during the intervention and comparison groups. When scheduling participants for follow-up visits, records will be reviewed to be sure that facilitators are not paired with their group participants.

Follow-up visits will be scheduled within a **two-week target window** based on participant availability and site capacity; visits may be scheduled either up to one week before or up to two weeks after the target follow-up date. Participants who miss an appointment within the target window or cannot complete the assessment at that time will be scheduled for an appointment outside of the target window, but within the acceptable window period. For the 3-month and 6-month visits, the acceptable window period will be one week before the start of the target window and two weeks after the target window.

1 Week

2 Weeks

2 weeks (3 & 6 month FU)

12 weeks (12 month FU)

**Target Window**

**Figure 1. Acceptable Follow-Up Assessment Window**

Every effort will be made by staff to ensure that participants are assessed in follow-up visits within, first, the target window, and second, the acceptable window. If it is not possible to assess within these windows, assessment will take place at whatever time possible with the primary intent of getting the participant back on track with their original 3, 6 and 12-month follow-up waves. A judgment call will be made as to how best to handle the situation by the staff given the particular circumstances for the individual participant and point in time that he contacted. For example, if a participant was out of the country during the 3-month window and eventually calls the site at the 5 month point, the staff person will assess participant willingness and ability to attend follow-up in the 6-month window and based on the individual participant either (a) schedule him for an assessment now and in a month, during the 6-month follow-up window (if the participant is willing and able to do both); (b) schedule him for the 6-months assessment only (if the participant is available then and unwilling to attend 2 follow-up assessments close to one another in the near future), or (c) schedule him for an immediate assessment only (if the other two options are not possible). This is done because data collected outside of a follow-up window are difficult to categorize for assessment purposes and thus are less desirable than data within 3, 6 and 12-month windows as defined earlier. For example, if data are collected at 5 months and at 6 months, the 6-month data will obviously be used for analyses in the 6-month wave, however the 5-month data will likely not be analyzed in the 3-month wave because (1) data were collected well after the 3-month wave and are actually closer in time to the 6-month wave, and (2) since we have 6 month data already, we won’t use the 5-month data (which also happens to overlap significantly with the 3-month behavioral assessment window). Alternatively, if data are collected at 5 months and it is not possible to assess the participant again during a 6 month wave, the 5-month data are preferred to no data at all. Thus, assessment of each individual situation and a judgment call are required of staff when participants are reached outside of a follow-up window. When a participant is assessed within the acceptable window for the 3-month assessment, there must be at least 90 days between the 3 and 6 month assessments.

For the 12-month assessment, there will be a two-week target window (one week before and after the 12-month date). The acceptable window for this final follow-up visit is longer, however: one week before the target window begins and 3 months after the target window ends. This final acceptable window is more generous because it is crucial that as many participants as possible participate in the final visit so that we can fully measure the potential impact of the intervention.

**9. Retention Efforts**

**Locator Information.** Participant locator and contact information (see below) will be collected and periodically updated in order to remind participants of their upcoming visits and enhance study retention. This information will be updated in a locator information database at the baseline visit, at the end of the intervention and at the follow-up visits (3, 6, and 12 months). In addition, contact will be made at 9 months to update locator information. Staff will update the database with the new locator information, but they will not share this information with any other person or organization.

Whenever the project staff uses this information to try to locate a participant, they will identify themselves as being from the organization conducting the study. If staff contact friends, family members, or another approved contact person, they will tell the contact person that the participant is in a research study and has provided written permission to contact this person or organization only to obtain locating information. Staff will not reveal to the contact person what the study is about (HIV prevention) or that the study involves substance use or MSM.

The comprehensive database tracking system will hold all personal information for each participant, including contact information and scheduled and completed appointments (including appointment times and dates). These data will remain separate from the assessment data to protect participant confidentiality. The database will be able to automatically generate the following items at any given time: a list of reminder cards to be sent to participants, a list of participants who need to be contacted via telephone or other method, and a schedule of upcoming appointments. These items will be generated and processed on at minimum a weekly basis throughout the study.

Obtaining reliable locator data at the baseline appointment is essential to contact at subsequent visits. Informed consent to participate in the study should include an explanation by the recruiter that they are agreeing to provide contact information and do their best to voluntarily attend all study visits. As mentioned earlier, a locator form will be used to collect data to allow study staff to contact the participant and remind them of their next visit. Participants without a stable email or postal address, or phone number, may drop in at the study office to set up an appointment or to complete the visit.

Data on the Locator Forms (see Appendix UU), which vary slightly by study sight based on local procedures, include participant name and study ID; date and location of recruitment; address, phone numbers [home, work, cell] and e-mail addresses to contact the subject for the next study visit; name, address, and phone number of friends, family members, or other people who are likely to know how to contact them over the next 15 months (their relationship to the participant will be noted). Contact information may specify whether or not project mail can be sent to this address, and whether or not it is okay to mention the name of the study in a message. Participants will be asked to suggest the best way to contact them (e.g., phone or email; time of day to call and which number to try first). The locator form asks other ways to reach the participant, including café, bar, or club; if it is okay to come by their residence and leave a message; and whether the participant has other ideas how they could be contacted. This information will be obtained on a purely voluntary basis (i.e., not a requirement for participation in the study). However, staff will make a judgment call as to whether or not limited contact information is adequate for participation given the individual circumstances and potential for successful staff follow-through with the participant. For example, if a person does not have a phone or email address but they have received services regularly at a known agency for an extended period of time and indicate that study staff may contact them through the agency, then given other circumstances for the individual (e.g., an address, contact information for others close to them) a study staff person may consider this adequate contact information for the individual to participate.

**Retention before and during the group sessions.** Because participants for each cohort will be recruited over several weeks, they will get a call or note from one of the project staff a few days before the first session. Participants will be asked at the end of the screening what would be the best way for us to remind them of the appointment. Some participants will have a phone and be willing to be called; others may wish to be contacted through e-mail. Those who do not prefer these modes of contact may be contacted through agencies where they receive services (by leaving a note) or through outreach workers going out into the communities where people live. In all cases, the participant will consent to be contacted in the manner used. If a participant consents to have a note left with agency staff, the note will be sealed. We will explain to participants that in all cases, a clear boundary exists between the research project and agency staff that prevents the sharing of information we learn about them during the research study with anyone at the agency.

Participant retention during the intervention will be enhanced in several ways. First, participants will be reimbursed for each session they attend (described earlier) to offset travel costs and for their time. At the beginning of each week, group leaders will give reminder calls to or send emails to remind them of that week’s sessions. In addition, if someone in either the intervention or comparison arm misses a session, the group facilitators will call or contact the participant to determine why a given session was missed and to try to encourage them to attend the next session. Participants will be asked to call the project if they are going to miss a session.

**Retention during the follow-up period.** Participant retention after the intervention will be enhanced in several ways. First, participants will be reimbursed $35, $40 ($25, $35 in NY) and $50 for 3, 6 and 12 month follow-up visits, respectively, to offset travel costs and time. The provision of a monetary reimbursement should enhance participation in the follow up visits. In addition, extensive procedures will be used to promote participant attendance at the follow-up visits. This is particularly important in an intervention trial, where high-levels of follow-up are required for examining the effectiveness of the intervention. At each follow-up visit (3, 6 and 12 months), and at 9 months after the final group session, subjects will be asked to update their locator information.

Letters will be sent out or phone calls will be made up to several weeks or more prior to the follow-up visit due date requesting that they contact the main office to confirm or reschedule an appointment, and reminder calls will be made within several days of the appointment. The letters (see Appendix UU) will thank the participants for being in the program, remind them of the date and time of their next follow-up appointment (in 3 or 6 months), and note the telephone number that can be called if rescheduling is necessary. The letters will be discrete with regard to the nature and purpose of the study. The letter will state that if they miss this visit, to please call to reschedule. Study staff will make three attempts over the window period (5 weeks for 3 and 6 month follow-ups; 15 weeks for 12-month follow-up) to contact participants who do not respond. Participants who do not respond and cannot be located during the acceptable window period are classified as having missed that visit. If staff are contacted by the participant after the acceptable assessment window period, a follow-up appointment will be scheduled nonetheless and the participant will be scheduled for the next follow-up visit according to their original follow-up schedule. Whenever possible, the next follow-up visit will be scheduled while the participant is in the office for the current visit. Once a participant is voluntarily enrolled in the study, staff will continue with contact attempts until a participant expresses the desire to be dropped from the study. For example, repeat no-shows within a particular follow-up window will continue to be rescheduled for follow-up until that particular window closes.

If a participant moves away from the original project city during the study, other arrangements will be made for follow-up. If the participant moves to a location close to another Project Mix site (i.e., Chicago, Los Angeles, New York City, San Francisco), project staff at the original city will attempt to gain approval from the participant to transfer contact and tracking information about the participant to the new city (see Appendix VV for release of information forms by site). The new city will need to receive informed consent from the participant, using the local consent form and consenting procedure. If written approval and consent are not obtained from the participant, or the participant moves to a location that is not close to one of the project cities, follow-up visits will be conducted over the phone by staff at the original project site. If the participant tested HIV-negative at baseline and moved to another project site during the study, the participant will be tested on site at 12-month follow-up as described in the protocol. If the participant tested HIV-negative at baseline and moved to a location other than a Project Mix site, the participant will not be asked to complete an HIV test as part of the study procedure. Similarly, safe and private alternative locations will be considered for follow-up assessment for local participants within a site who are unable to come to the office.

**Ascertaining reason for losses to follow-up.** A list of the study ID’s of lost participants will be maintained at each site, with disposition being recorded on the list when it is determined. In some cases, the study staff may learn that the participant has moved away; staff will continue to attempt to reach these participants for follow-up and make every reasonable effort to accommodate the participant in order to complete the follow-up assessment. Options for long-distance follow-up include staff-administered ACASI assessment over the phone; assessment coordinated through another Project Mix study site if the participant moved to a project city (Chicago, Los Angeles, New York, San Francisco). Relocation information may be provided by the participant, his or her contacts, or by the US Postal Service. Study letters sent to participants will be stamped with “forwarding and address correction requested”. This service is provided for 50 cents. At the end of each year of follow-up, sites may consult local or national death records or indexes to determine whether lost study participants have died.

Participants who are in substance abuse treatment or prison will be classified as such. Subjects may also refuse to complete any follow-up visits; these will not be combined with study losses, but classified as refusals. For some lost subjects, reason for loss will not be determined. At the end of the study, follow-up rates will be calculated.

**C. Logistics and Reimbursement**

## 1. Time

The baseline behavioral assessment session will take approximately 2.5 hours (range, 2-3 hours) to complete, including written consenting procedures, the ACASI survey, and HIV counseling and testing. Each of the six sessions for the intervention and comparison arms will be 2 hours long, and each of the three follow-up assessment sessions (at 3, 6 and 12 months post intervention) will be approximately 2 hours long.

## 2. Reimbursement

At the end of each appointment or session, participants will be reimbursed for their time and effort. Individual reimbursement will be $40 for the baseline appointment in Chicago and San Francisco ($25 in New York; $35 in Los Angeles); $25 for each intervention session in Chicago and New York ($35 in Los Angeles and San Francisco); and $35, $40 ($25, $35 in NY) and $50 for each of the three follow-up assessment visits (at 3, 6, and 12 months). Sites determined their own reimbursement rates given their extensive experience with the study population in their locales. Rates are comparable to other similar studies implemented by the PIs as well as reimbursement rates used in this study during the earlier focus group and pilot phases. Rates take into account transportation and time costs, and the local cost of living.

As described earlier, participants who arrive too late to complete the baseline assessment session, depending on staff availability, will not receive reimbursement and, if possible, they will be rescheduled. Similarly, if participants arrive too late to the first group session to be randomized into an intervention or comparison group (approximately 15 minutes after the scheduled appointment time), they will not receive compensation; however, they will be rescheduled for another appointment if possible.

If less than ten participants (five per arm) show up for the first group session appointment, the men who show up will be reimbursed $15 for their time and the group will be rescheduled for another time. If more than 20 participants show up for the first group session (10 per arm), those who arrive after the first 20 men will receive $15 for their time and will be rescheduled for another appointment if possible. Persons who are eligible at the first full screening (Screener 1) but are ineligible on confirmatory screening at the baseline appointment will be thanked, reimbursed $15 for their time, and placed on a wait list for later contact and re-screening if they so desire.

This procedure will be explained to eligible participants during screening and at the baseline assessment individual session. If participants arrive more than 30 minutes after Sessions 2-6 start, they will be encouraged to participate in the group but will not receive a reimbursement for that session.

# The maximum amount of reimbursement a participant could receive for successfully completing the full trial is $285 in New York, $315 in Chicago, $375 in San Francisco, and $370 in Los Angeles.

Reimbursement distribution will be documented at each site using either a receipt system, a centralized reimbursement log or both. The participant and staff person will both initial the receipt and/or the log upon reimbursement distribution, citing the date, time, location and specific purpose (e.g., session number that was completed, baseline assessment, Session 1 overflow) for which the reimbursement is distributed. The reimbursement receipts and/or log, and the remaining cash on hand, will be balanced daily and weekly by project staff to ensure financial security and integrity.

# D. Risks and Benefits

## 1. Risk to Participants

Enrollment in the behavioral intervention trial involves minimal risk to participants. The voluntary informed consent form and process make clear all of the potential risks of study participation (Appendices KK through NN by site). Participants will feel a slight pain in their finger when they receive the needle stick for rapid HIV testing. Staff will be well trained in HIV testing and referral procedures as required by the CDC and their states. Participants newly diagnosed as HIV-positive will receive counseling and referrals as outlined by CDC, also covered well by federal and state required training. Some participants may feel uncomfortable talking in a group setting with strangers. Participants experiencing significant distress or requesting services will be assisted immediately and referred to the appropriate supportive services.

Participants will be informed that, according to the law, staff must report some illegal behaviors to state or local authorities. These laws are complex and vary by state (CA, IL, NY); see consent forms for specific laws by site (Appendices KK-NN). In general, however, staff must report (1) if a person indicates that he intends to harm himself or others, (2) an elder or dependent person is being physically or sexually abused, (3) someone under age 18 is being physically or sexually abused (by a guardian/caregiver in NY and IL), and (4) in CA, having (a) any consensual sexual contact with a minor under age 14, (b) any consensual sexual contact with a minor age 14 or 15 and the adult is at least 10 years older, and (c) consensual sexual intercourse with a person under age 16 and the adult is age 21 or older.

## 2. Confidentiality

The researchers and project staff will keep personal information confidential, including HIV status. Confidentiality and scientific ethics will be covered during staff training to emphasize the importance of the issue. Office procedures are also put in place to maintain sensitive information (e.g., contact information, HIV status) under lock and key. For example, HIV testing results and behavioral data will be associated with an ID number and not a name, and contact information is kept in separate files from data gathered during the study. A limited number of staff will have keys and could access to those files. Additionally, when HIV results are reporting to local health departments (as in San Francisco and Los Angeles), no identifying information is used.

Complete privacy, however, cannot be absolutely guaranteed because there is potential risk that information shared by a participant during a group session could be discussed by another participant outside of the group. We have taken steps to minimize the chance of a breach of confidentiality. The consent form emphasizes that information about other participants should not be shared with others, particularly with people outside of the group; facilitators will highlight those points during the informed consent process at the beginning of the first group session, and throughout the group sessions. Further, it will be emphasized with participants during the consenting procedure and in the group sessions not to reveal personal identifiers or discuss other identifying information about third parties (e.g., sex partners of other men in their group) both in and outside of the group. Individuals will be instructed that they do not have to disclose personal information that they are uncomfortable sharing.

## 3. Benefits

Few direct benefits to participants are anticipated in this phase of the research. However, the participants may feel good about themselves as a result of helping researchers address issues related to health promotion and HIV prevention for SUMSM, and they may appreciate interactions with other MSM. It is possible that some individuals may gain information, skills and insight through the group session experience that could potentially improve their lives.

**E. Data Management and Analyses**

## 1. Data Management

Study forms, site reports, and audio tapes will be kept in locked file cabinets at the project sites and at CDC for the duration of the study.

Quantitative data (ACASI survey and HIV test results) will be stored only with an ID and not with a direct personal identifier such as name or phone number; personal identifying information will be kept locked in project files and a limited number of project staff will have access to keys to the files. A participant database containing contact information and IDs will be double password protected with access only to key project staff. Again, there are no personal identifiers directly associated with any data other than the contact information files. Contact information will be destroyed six months after completion of the study unless participants indicate that project staff may keep it on file for future studies or programs, or they request a copy of the primary results of this study. However, six months after the study is completed, study ID numbers for all participants will be de-linked from contact information in the participant database. Audio tapes will be destroyed 2 years after the end of the study (i.e., two years after the last follow-up assessment is completed).

Each site will collect data from screening and baseline interviews on computers designated for data collection. Each day, the interviews on the data collection computers will be zipped, backed-up and moved to the Data Manager’s computer by disk. These daily data transfer disks will then be locked in file cabinets for back-up. Data will be deleted from the data collection computers as soon as the data is successfully transferred to disk and to the Data Manager’s computer. Weekly, new data on the Data Manager’s computer will be encrypted and uploaded to CDC via a secure data network based at CDC. Personal identifiers will **not** be sent to CDC. Management and cleaning of the cross-site data set will occur at CDC.

## 2. Data Analyses

### *a) Qualitative data.*

Observer/reviewer brief reports for each session will be compiled into a summary report when periodic observations or audio- reviews of sessions are made for program quality assurance (approximately 10% of sessions). Summary reports will be reviewed by site and CDC staff and discussed; these reports will be used as a reference and basis for additional training of facilitators if needed and to ensure appropriate implementation of the program. Further assessment of tapes (e.g., content analysis) may be undertaken to better understand behavior and motivations of participants and the potential effectiveness of study materials on behavior change.

*b) Quantitative data.*

Data will be cleaned and merged centrally at CDC. Primary outcomes measured at 3, 6 and 12 months will be analyzed for differences between intervention and comparison groups; repeated measure analyses will be conducted across waves. Behavioral outcomes will be analyzed for overall intervention effects across follow-up visits as well as for time-treatment interactions. Baseline behavior, demographic variables, and other potential cofactors will be included in multivariate models. Psychosocial and other potential mediators of sexual risk behavior will be examined. City and group cohort will also be examined as potential confounding variables and treated accordingly in analysis.

## 3. Information Security

During recruiting and screening, names and telephone numbers of potential and actual participants will be collected to facilitate participation. These data will be kept in a separate, locked file cabinet to which only staff with a work-related need will have access. Daily transfer/back-up data disks will also be kept under lock and key. A participant database containing contact information and ID numbers (computer-generated at Screener 1) will be double password protected with access only to key project staff. Each site will keep to a realistic minimum the number of staff with access to this information and these files. A list of pilot participants will be checked during trial enrollment. This step is taken to prevent pilot participants from involvement in the full intervention trial, however focus group participants are eligible to participate in the intervention trial. All contact information will be kept until six months after completion of the study unless participants indicate that project staff may keep it on file for future studies or program, or they request a copy of the primary results of this study. However to ensure information security, study ID numbers for all participants will be de-linked from contact information in the participant database six months after the study ends.

## 4. Dissemination, Notification, and Reporting of Results

### a) Notifying participants of individual results and study findings.

Participants who indicate that they want to receive a summary of primary findings from the trial will be sent a summary of general findings at the time they become available for dissemination. Individual results of ACASI data will not be analyzed or distributed. HIV testing results for those who are tested in the study (i.e., men who had not tested positive at an earlier date or who had but could not provide such documentation) will be provided to the individual at the time of counseling and testing (baseline and 12-month follow-up) and according to the standard of care outlined earlier.

### b) Dissemination of findings.

Participants who indicate to staff (via locator information form data) that they want to receive a summary of the primary results of the study will receive a mailing when that summary is available. Study results will be submitted for peer review by professional journals and conference organizers. Dissemination of findings to local communities will take place through community forums, agency meetings, and the media.

## References

Centers for Disease Control and Prevention (2001). Revised guidelines for HIV

counseling, testing and referral. MMWR, 50 (No. RR-19), 1-58.

Centers for Disease Control and Prevention. (2002). HIV/AIDS Surveillance Report, 2001, 13(2).

Chesney, M. A., Barrett, D. C., & Stall, R. (1998). Histories of substance use and risk behavior: precursors to HIV seroconversion in homosexual men. Am J Public Health, 88(1), 113-6.

Colfax, G. N. , Mansergh, G., Guzman, R., Vittinghoff, E., Marks, G., Rader, M., & Buchbinder, S. (2001). Drug use and sexual risk behavior among gay and bisexual men who attend circuit parties: a venue-based comparison. J Acquir Immune Defic Syndr, 28(4), 373-9.

Greene, G. W., Rossi, S. R., Rossi, J. S., Velicer, W. F., Rossi, J. S., Fava, J. L., & Prochaska, J. O. (1999). Dietary applications of the stages of change model. J Amer Dietetic Assoc, 99, 673–678.

Halkitis, P. N., Parsons, J. T., & Stirratt, M. J. (2001). A double epidemic: crystal methamphetamine drug use in relation to HIV transmission among gay men. J of Homosexuality, 41( 2), 17-35.

Johnson, W. D., Hedges, L., Ramirez, G., & et al. (2002). HIV prevention research for men who have sex with men: a systematic review and meta-analysis. J of Acquired Immune Deficiency Syndrome, 30 (Suppl 1), S118-S129.

Kalichman, S. C., Weinhardt, L., KiFonzo, K., Austin, J., & Luke, W. (2002). Sensation seeking and alcohol use as markers of sexual transmission risk behavior in HIV-positive men. Ann Behav Med, 24(3), 229-235.

Lewis, L. A., & Ross, M. W. (1995). The gay dance party culture in Sydney: a qualitative analysis. J of Homosexuality, 29, 41-70.

Mansergh, G., Colfax, G. N., Marks, G., Rader, M., Guzman, R., & Buchbinder, S. (2001). The Circuit Party Men's Health Survey: findings and implications for gay and bisexual men. Am J Public Health, 91(6), 953-8.

Marcus, B. H., Bock, B. C., Pinto, B. M., Forsyth, L. H., Roberts, M. B., & Traficante, R. M. (1998). Efficacy of an individualized, motivationally-tailored physical activity intervention. Annals Behav Med, 20, 174–180.

McKirnan, D. J., Vanable, P. A., Ostrow, D. G., & Hope, B. (2001). Expectancies of sexual "escape" and sexual risk among drug and alcohol- involved gay and bisexual men. J Subst Abuse, 13(1-2), 137-54.

Prochaska, J. O., DiClemente, C. C., Velicer, W. F., & Rossi, J. S. (1993). Standardized, individualized, interactive, and personalized self-help programs for smoking cessation. Health Psychology, 12, 399–405.

Purcell, D. W., Parsons, J. T., Halkitis, P. N., Mizuno, Y., & Woods, W. J. (2001). Substance use and sexual transmission risk behavior of HIV-positive men who have sex with men. J Subst Abuse, 13(1-2), 185-200.

Rakowski, W., Ehrich, B., Goldstein, M. G., Rimer, B. K., Pearlman, D. N., Clark, M. A., Velicer, W. F., & Woolverton, H. (1998). Increasing mammography screening among women aged 40–74 by use of a stage-matched, tailored intervention. Preventive Med, 27, 748–756.

Reback, C. J., & Grella, C. E. (1999). HIV risk behaviors of gay and bisexual male methamphetamine users contacted through street outreach. Journal of Drug Issues, 29(1), 155-166.

Seage III, G. R., Mayer, K. H., Wold, C., Lenderking, W. R., Goldstein, R., Cai, B., Gross, M., Heeren, T., & Hingson, R. (1998). The social context of drinking, drug use, and unsafe sex in the Boston Young Men Study. J Acquir Immune Defic Syndr Hum Retrovirol, 17(4), 368-75.

Stall, R., Paul, J. P., Greenwood, G., Pollack, L. M., Bein, E., Crosby, G. M., Mills, T. C., Binson, D., Coates, T. J., & Catania, J. A. (2001). Alcohol use, drug use and alcohol-related problems among men who have sex with men: the Urban Men's Health Study. Addiction, 96(11), 1589-601.
